# MOMP from *Campylobacter jejuni* Is a Trimer of 18-Stranded β-Barrel Monomers with a Ca^2 +^ Ion Bound at the Constriction Zone

**DOI:** 10.1016/j.jmb.2016.09.021

**Published:** 2016-11-06

**Authors:** Luana G M Ferrara, Gregor D Wallat, Lucile Moynié, Naresh N Dhanasekar, Soumeya Aliouane, Silvia Acosta-Gutiérrez, Jean-Marie Pagès, Jean-Michel Bolla, Mathias Winterhalter, Matteo Ceccarelli, James H Naismith

**Affiliations:** 1 Biomedical Sciences Research Complex, University of St Andrews, 09042 St Andrews, UK; 2 Department of Life Sciences and Chemistry, Jacobs University Bremen, 28719 Bremen, Germany; 3 Aix Marseille Univ, IRBA, TMCD2, 13385 Marseille, France; 4 Department of Physics, University of Cagliari, Cittadella Universitaria Monserrato, S.P8-km 0.700, 09042 Monserrato, Cagliari (CA), Italy; 5 State Key Laboratory of Biotherapy, Sichuan University, Chengdu 610041, China

**Keywords:** MOMP, major outer membrane protein, SeMet, selenomethionine, rMOMP, high-resolution structure of MOMP purified from an *E. coli* overexpression system, nMOMP, high-resolution structure of MOMP purified from *Campylobacter*, PEG, polyethylene glycol, RMSF, root mean square fluctuations, MD, molecular dynamics, Octyl-POE, *n*-octylpolyoxyethylene, CV, column volume, *Campylobacter*, outer membrane proteins, antibiotic resistance, β-barrel, porins

## Abstract

The Gram-negative organism *Campylobacter jejuni* is the major cause of food poisoning. Unlike *Escherichia coli*, which has two major porins, OmpC and OmpF, *C. jejuni* has one, termed major outer membrane protein (MOMP) through which nutrients and antibiotics transit. We report the 2.1-Å crystal structure of *C. jejuni* MOMP expressed in *E. coli* and a lower resolution but otherwise identical structure purified directly from *C. jejuni.* The 2.1-Å resolution structure of recombinant MOMP showed that although the protein has timeric arrangement similar to OmpC, it is an 18-stranded, not 16-stranded, β-barrel. The structure has identified a Ca^2 +^ bound at the constriction zone, which is functionally significant as suggested by molecular dynamics and single-channel experiments. The water-filled channel of MOMP has a narrow constriction zone, and single-molecule studies show a monomeric conductivity of 0.7 ± 0.2 nS and a trimeric conductance of 2.2 ± 0.2 nS. The ion neutralizes negative charges at the constriction zone, reducing the transverse electric field and reversing ion selectivity. Modeling of the transit of ciprofloxacin, an antibiotic of choice for treating *Campylobacter* infection, through the pore of MOMP reveals a trajectory that is dependent upon the presence metal ion.

## Highlights

• The crystal structure of MOMP, the general diffusion porin of Campylobacter, has been determined.
• The protein is an 18-stranded β-barrel that is different than the 16-stranded OmpC and OmpF proteins from *E. coli*, but like them, MOMP is trimeric.
• The protein has a central pore size and conductivity intermediate between OmpC and OmpF.
• A Ca^2 +^ ion bound at the constriction zone influences the biophysical properties of porin.
• The trajectory of the transit of the antibiotic ciprofloxacin through the pore is dependent on the presence of a metal ion.

## Graphical Abstract

**Figure:**
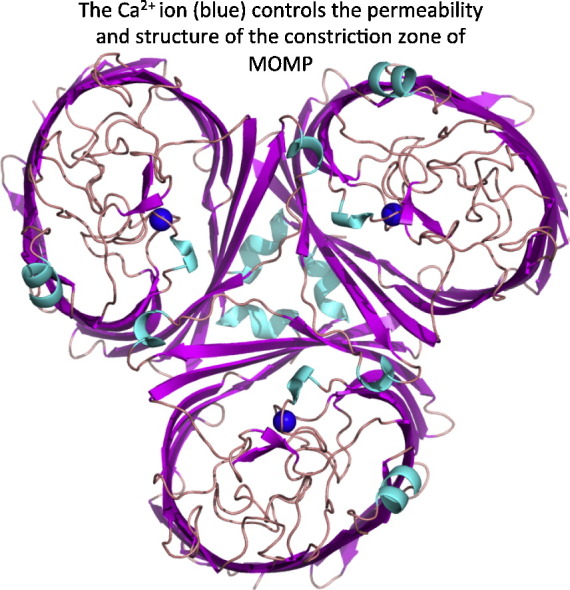

## Introduction

*Campylobacter* are Gram-negative bacteria belonging to the ε-proteobacteria, which are commensal in livestock, notably in poultry, and are thought to be the main natural reservoir. Human infection most commonly occurs via consumption of undercooked animal products or contaminated water and through contact with animals [1]. The pathogen *Campylobacter jejuni* is the major cause of human food poisoning and represents an economic burden [2]. In general, *C. jejuni* infection resolves without antibiotic treatment, but more serious cases (usually in infants) can be treated straightforwardly with antibiotics. Untreated campylobacteriosis can lead to a cycle of recurrent infections, and this in turn progresses to irritable bowel syndrome [3]. In rare but serious cases, infected individuals can develop non-trauma-related paralysis through Guillain–Barré syndrome [4].

In the recent years, multi-antibiotic-resistant *Campylobacter* species have emerged, and although not common, this trend has alarmed health care professionals [5]. Bacteria resist antibiotics by the expression of drug-modifying enzymes (e.g., β-lactamase), mutation of target (ribosome), drug efflux pumps (e.g., AcrAB-TolC in Enterobacteria, Mex family in Pseudomonads, and CmeABC in Campylobacterales), and reduced uptake (e.g., decreased expression or mutations of porins in Gram-negative bacteria) [6], [7]. Porins are responsible for the uptake of nutrients through the outer membrane of the Gram-negative bacteria by passive diffusion along the concentration gradients. Their substrate uptake can be specific or non-specific, depending on the nature of the protein [7].

Most antibiotics cannot pass through the outer membrane itself so instead must enter the cell via non-specific porins. In *Escherichia coli*, two outer membrane proteins, OmpC and OmpF, are predominant. A shift in expression profile from OmpF (higher conductance) to OmpC (lower conductance and less permeable to antibiotics) has been commonly observed in drug-resistant clinical isolates [8]. Combined structural, physical, and computational studies of a series of clinical OmpC mutations revealed a molecular basis for altered antibiotic uptake [9], [10]. In contrast to *E. coli*, *C. jejuni* has only one major outer membrane protein (MOMP), which is thus far present in all isolates and is highly (but not absolutely) conserved in other Campylobacteria. MOMP is a 44-kDa protein, which has the sequence signature typical of β-barrel porin, and CD spectroscopy [11] confirmed the predominance of β-strand typical of porins. MOMP has been reported to be critical to the stability and integrity of the outer membrane of *C. jejuni* [12], while other studies have shown MOMP to have a role in adhesion to epithelial cells [13]. As might be expected, given its location in the bacterial cell, antibodies are frequently raised against it by the human immune system [14], [15], [16], and this has led to ideas for vaccine development [17]. Structural and biophysical data are thus highly desired for this protein.

Here, we report the successful recombinant expression, extraction, purification, and structure determination of *C. jejuni* 85H strain MOMP from an *E. coli* overexpression system*. Campylobacter* are ε-proteobacteria, while *E. coli* belongs to the γ-proteobacteria, meaning there are large differences in lipid composition in the outer membrane. To allay concerns about the “nativeness” of this recombinant structure arising from the difference in the lipid environment during expression, we also determined the structure of MOMP purified directly from *Campylobacter* and we found it to be essentially identical. Crystals, but no structure of MOMP from *C. jejuni*, have been previously reported [18]. The lower purity of the MOMP obtained directly from *Campylobacter* highlighted the key advantage of *E. coli* expression. MOMP exhibits conductivity similar to OmpC; however, its constriction zone has a number of important differences that may relate to antibiotic permeability. Using this structure, accurate models can be generated for MOMPs from other strains and other species of *Campylobacter*.

## Results

### Structure of MOMP

Codon-optimized MOMP-coding gene from strain 85H was cloned in pTAMAHisTEV vector, which inserts a tobacco etch virus and a cleavable hexa-histidine tag between the protein and the TamA signal peptide. After the overexpression in *E. coli* and purification, a final yield of 12 mg of MOMP per 50 g of *E. coli* cell paste was obtained. In a different approach, native MOMP was purified from *Campylobacter* as described previously [11] and yielded 1 mg protein per 3 g of *C. jejuni* cell paste. Both proteins gave crystals, although in different conditions with different crystal symmetry.

The structure of the overexpressed selenomethionine (SeMet) variant MOMP was solved using single-wavelength anomalous diffraction to provide initial phases, which were improved by density modification. This structure was then used to solve the high-resolution structure of MOMP purified from an *E. coli* overexpression system (rMOMP). The protein purified from *Campylobacter* (nMOMP) was solved via molecular replacement using the recombinant MOMP structure as a search model (Table 1). Beyond small differences due to crystal packing and resolution, we have not detected any meaningful structural difference between the two proteins (RMSD of 0.45 Å for 398 Cα position using SSM [19]; Supplementary Fig. 1). Our discussion focuses on the higher-resolution rMOMP structure (2.1 Å). We use rMOMP and nMOMP, where we discuss the evaluation of each preparation method (to establish they are essentially identical), and MOMP to refer to the generic protein.

**Fig. 1:**
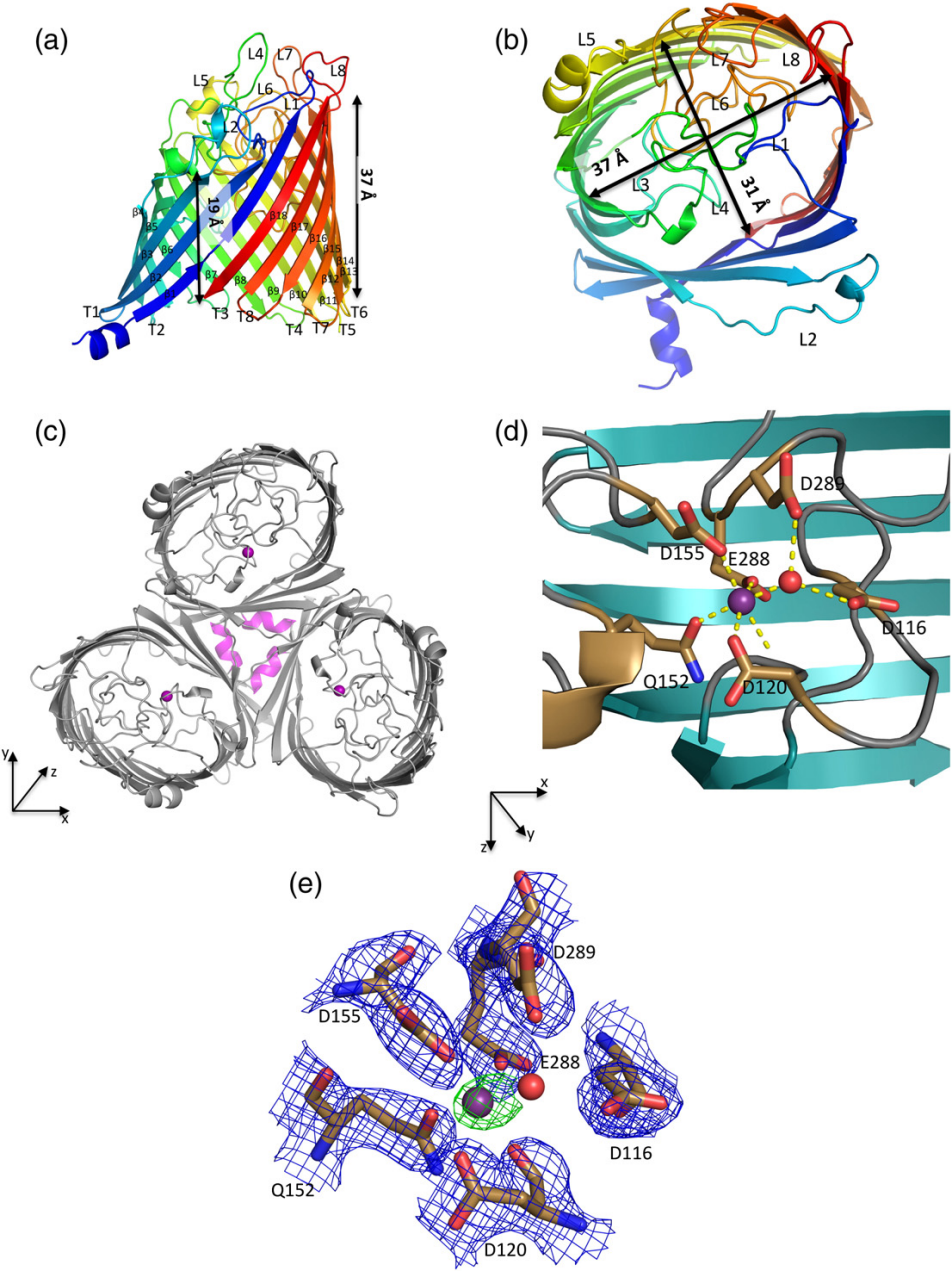
The structure of the MOMP monomer. (a) Viewed from the side, parallel to the membrane. (b) The structure has been rotated 90° so that it is viewed from the outside of the cell (looking in), perpendicular to the membrane. (c) The trimer viewed as in (b), the periplasmic N-terminal α-helix is colored magenta and the calcium in purple. An XYZ axis is shown to orientation. (d) The calcium is depicted as a purple sphere and the water molecule as a red sphere. Residues involved in the calcium coordination are shown as sticks. An XYZ axis shows the view that has been rotated by 90° around the *X* axis when compared to (c). (e) A detailed view of the amino acids involved in calcium-binding site; same orientation as in (d). The F_o_-F_c_ and 2F_o_-F_c_ electron density maps at 5σ and 2σ, respectively, have shown the final refined coordinates. The phases for the calculation of the map were based on a model that had never included the metal ion.

MOMP is an 18-stranded antiparallel β-barrel porin with an elliptical shape common to other 18-stranded porins. The signal peptide of nMOMP from *C. jejuni* strain 85H (uniprot entry number: Q659I5) was predicted to cleave between Ala22 and Thr23. Henceforth, Thr23 being the first residue in the tertiary structure was defined as the first residue in our residue count and was renamed to Thr1. In rMOMP, however, due to cloning artifacts, we have three additional non-native residues after signal peptide cleavage (Gly-Ala-Met), followed by a single point mutation of Thr1 to Gly. Typical of porins, the axis of the strands is offset, relative to the membrane normal. The long axis of the ellipse is approximately 37 Å (the distance from Gly179 to Glu280) and the minor axis is 31 Å (Arg45 to Ala234). The vertical height of the barrel varies between 19 Å (distance between Val10 and Phe86) and 37 Å (distance between Thr221 and Val334, on the opposite side; Fig. 1a–b). The N terminus on the periplasmic face has a strand, followed by an N-terminal α-helix that points away from the barrel wall (Fig. 1c). In the protein purified from *Campylobacter,* the helix is also present and adopts the same organization but lacks the short strand seen in the overexpressed recombinant protein. In keeping with convention for porins, extracellular loops connecting the strands are numbered sequentially and prefixed by L, whereas periplasmic loop connections are prefixed by T (also numbered sequentially). Four extracellular loops L1, L3, L4, and L6 fold inside of the barrel, with L3, L4, and L6 forming the constriction zone. Loop 4 contains Phe173-Lys174, the site of the proteinase K cleavage [20]; the structure shows that the site to be exposed is consistent with its cleavage. MOMP from another strain (79AH) is not cleaved at this position as it lacks the protease site. We observed additional electron density adjacent to L3 and L4 that we identify as a Ca^2 +^ (rather than Zn^2 +^ or Mg^2^^+^, or K^+^ or Na^+^) based on electron density and coordination (distance and geometry). The proteins expressed in *E. coli* have not had any Ca^2 +^ ion added and we conclude that the ion was bound during expression in *E. coli* and has remained attached in throughput purification. The ion has octahedral coordination, with one oxygen atom from the side chain of Asp120, Gln152, Asp155, and Glu288, and has the carbonyl of Asp120 with a water molecule filling the final sixth position. The water is in turn coordinated by the amino acid Asp116 and Asp289 (Fig. 1d–e). The metal ion creates a cross-link among L3, L4, and L6. Additionally, the difference electron density map showed a residual elongated density that was modeled as an ethylene glycol like the molecule [from polyethylene glycol (PEG) 400 or C8E4] in the rMOMP. This molecule sits below the constriction zone underneath L3. The protein purified from *Campylobacter* was crystallized in the presence of Ca^2 +^ ions and has an ion in an identical position but also has a second ion located outside the barrel at the interface between subunits. This ion is coordinated by Asp145 (main chain and side chain), Asn180 (side chain), and the main of Gly72 from the neighboring subunit. The recombinant structure shows a different side-chain conformation, implying that in the recombinant protein, this site does not exist. It had been hypothesized, according to molecular modeling, that in the 79AH strain, there is a metal-ion-binding site at Ser81 and Asp82 (found as Glu82 and Lys83 in 85H) that is important for the trimerization of 79AH [20]. These residues are located within a regular strand (β4); consequently, the side chains point in opposite directions and we suggest that there is no such metal-ion-binding site.

**Fig. 2:**
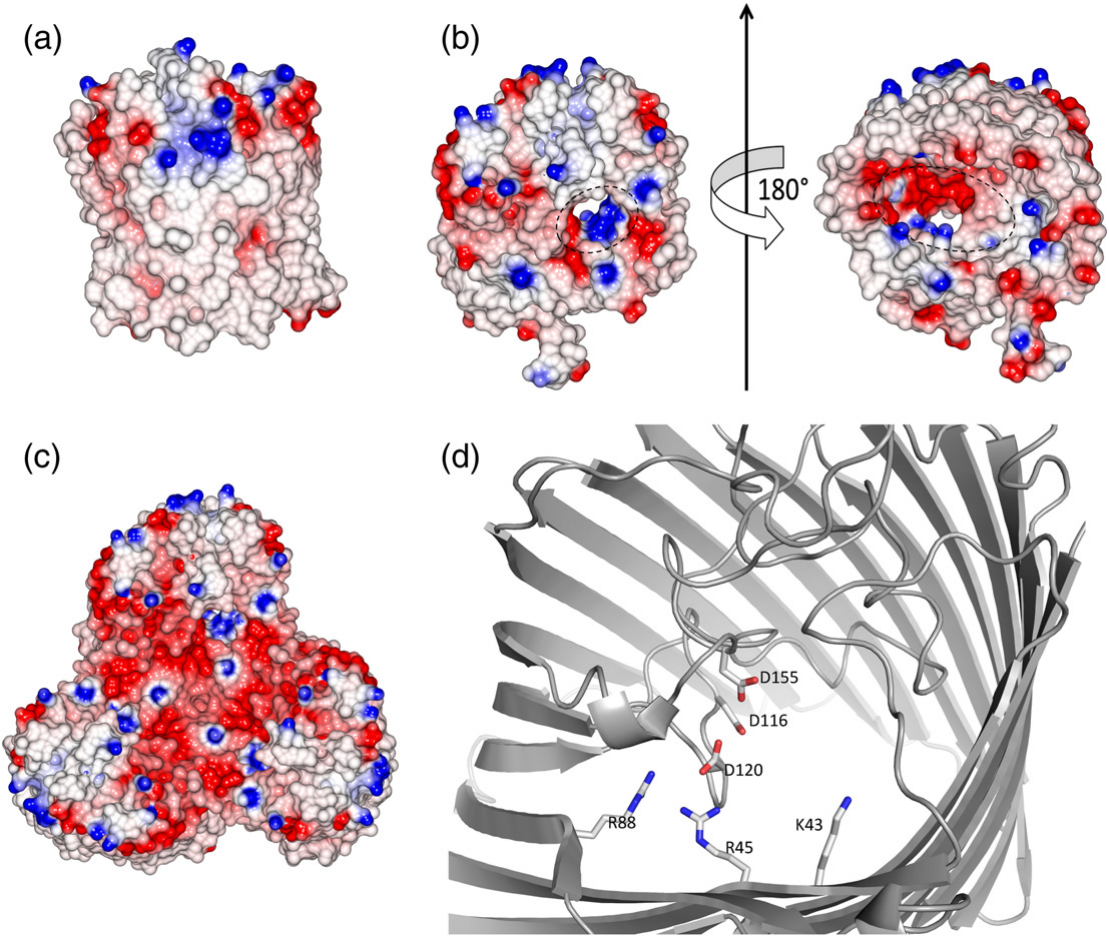
The pore of MOMP. (a) The surface of MOMP is colored by electrostatic charge calculated in CCP4MG [21]. The structure has the same orientation as in Fig. 1a. (b) The structure is colored as in (a), with the constriction zone circled. The structure on the left is the same orientation as in Fig. 1b; the structure on the right has been rotated 180° (in effect, looking from the periplasm through the membrane to outside the cell). (c) MOMP trimer viewed from outside the cell; the structure has the same orientation as in Fig. 1c. (d) A detailed view of the charged amino acids at the constriction zone; the view is from outside the cell and the same as in Fig. 1b. The Ca^2 +^ ion, which has been omitted for clarity, is bound to Glu155.

Analysis of the molecular surface using the program CCP4MG [21] shows that the outside wall of the barrel is uncharged as expected for proteins embedded within a hydrophobic lipid membrane bilayer. The extracellular surface of MOMP has regions of both positive and negative charge with the funnel-shaped region leading to the constriction zone, termed the eyelet, which is strongly negatively charged. The periplasmic face of the protein, including the inside surface of the barrel below the constriction, is predominantly negatively charged (Fig. 2). The pore axis is slightly offset, relative to the membrane normal. When viewed from the extracellular face, L7 sits above the entrance. Analysis with MolAxis [22] reveals that the constriction zone narrows to a radius of 2.6 Å. The constriction zone, like the protein, has an elliptical, not circular, shape. On one surface of the constriction zone, Tyr304 sits among amino acids Asp116, Asp120, Asp155, Asp289, and Glu300, which form a strongly negatively charged region of the pore; significantly, these residues also coordinate the Ca^2 +^ ion. On the opposite side of the constriction zone, the surface is formed by residues Arg17, Arg19, Arg45, and Arg398, which create a strongly positively charged region. This arrangement gives rise to a profound dipole across the short axis of the elliptical constriction zone, termed the transverse electric field. Lys43 and Lys404 are not in the constriction zone but point toward it from the extracellular and periplasmic sides, respectively. In addition to the metal ion, several contacts appear crucial to maintain the structure of the constriction zone; Arg362 makes salt bridges with Asp337 and Glu109; Arg45 with Asp65, Asp120, and Glu151.

Although only one monomer is present in the asymmetric unit, analysis of the crystal packing reveals a trimeric arrangement reminiscent of that seen in OmpC [23] and OmpF [24]. The identical trimeric arrangement is found in the protein purified from *Campylobacter*; in this case, the trimer is found in the crystallographic asymmetric unit. Analysis of the contacts between monomers using the PISA server [25] (which assesses the likely stability of multimers) identifies this trimer as the stable unit of MOMP. Gel-filtration data point to a trimeric arrangement consistent with electron microscopy of MOMP reconstituted into lipid bilayers [26]. The N-terminal Thr1 (Gly1 in recombinant MOMP) makes hydrogen bonds to strand 1 of the neighboring monomer; consequently, the three N-terminal helices in the trimer form a triangle. The main points of contact that stabilize the trimer arise from interactions with L2 from one monomer with L2, strand 5, strand 6, and L3 from the other monomer. Notably, residues Asn80 to Lys84 in this loop make main chain and, in some cases, side-chain hydrogen bonds across this interface. There is also a smaller area of contact on the periplasmic face between T8 (Lys370 to Phe376) on one monomer and T1 (Asp56 to Phe58) from the other. Each monomer–monomer interface buries over 2400 Å^2^ of surface area; the trimer buries 11,600 Å^2^ of surface area.

### Single-channel conductance measurements

**Fig. 3:**
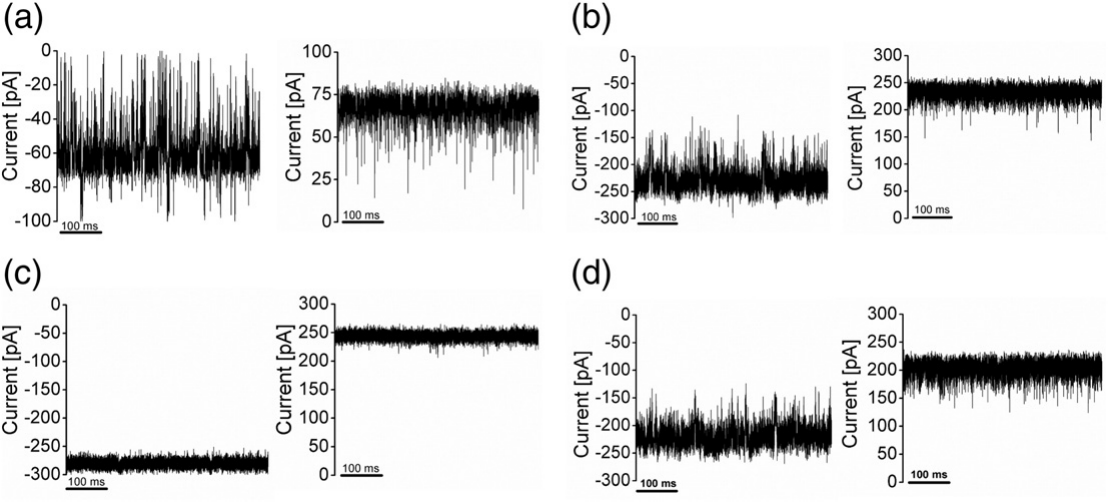
Representative ion-current traces of rMOMP in 1 M KCl and 10 mM Mes (pH 6.0). (a) The ion-current corresponding to one open monomer at negative (top panel, left) and positive (top panel, right) transmembrane potential of 100 mV. (b) The ion-current corresponding to the trimer at negative (top panel, left) and positive (top panel, right) transmembrane potential of 100 mV. (c) The ion-current corresponding to a trimer following the addition of 10 mM CaCl_2_ (1 M KCl at pH 6.0) at negative (bottom panel, left) and positive (bottom panel, right) transmembrane potential of 100 mV. (d) The ion-current corresponding to a trimer following the addition of 10 mM MgCl_2_ (1 M KCl at pH 6.0) at negative (bottom panel, left) and positive (bottom panel, right) transmembrane potential of 100 mV. The monomer and the trimer were produced from different bilayer measurements.

The pore-forming activities of both the rMOMP (Fig. 3) and nMOMP (Supplementary Fig. 3) were measured by single-channel ion-conductance measurements. Figure 3a and b shows the typical ion-current electrical signature of single monomeric and trimeric rMOMP, respectively. Figure 3a shows the monomers at negative (− 100 mV) and at positive (+ 100 mV) applied transmembrane voltages. It is interesting to note that at positive voltages, we observe only downward but never upward spikes, suggesting the presence of a monomer channel as opposed to the partially blocked oligomers. At negative voltages, the MOMP monomer has upward spikes leading to the closure of the pore (Fig. 3a and Supplementary Fig. 3b). After the single-channel insertion, we flush the cuvette intensively with fresh buffer to avoid further insertion. Figure 3b shows the typical traces of trimers. At positive voltages, the trimeric channel gives a smooth response, whereas at negative voltages, flickering noise occurs. The analysis of repeated single rMOMP channel reconstitution in 1 M KCl revealed two main conductance levels, a lower level of 0.7 ± 0.2 and a higher level of 2.2 ± 0.2 nS, suggesting a monomeric and trimeric state. The same analysis for nMOMP yields 0.7 ± 0.2 and 2.3 ± 0.3 nS, respectively. Like other trimeric porins, both monomer and trimer follow an ohmic conductance pattern between ± 200 mV (Supplementary Fig. 3a). No meaningful differences between the nMOMP and rMOMP proteins were observed. To investigate the homogeneity in the insertion of proteins, we followed multichannel measurements, and the distribution between monomers and trimers was random (Supplementary Fig. 3f). Control measurements with 1 M NaCl instead of KCl and 10 mM Mes (pH 6.0) revealed similar flickering at negative voltages (Supplementary Fig. 4a).

To elucidate the effect of divalent cation, we performed conductance measurements with 10 mM calcium chloride in the presence of 1 M KCl at pH 6.0. The presence of calcium chloride (Fig. 3c) eliminated the noise with a slight increase in conductance of 2.4 ± 0.2 nS at positive transmembrane potential of + 100 mV and a somewhat higher conductance of 2.8 ± 0.2 nS at negative transmembrane potential of − 100 mV. It is interesting to note that the measurements performed in either 10 mM MgCl_2_ (Fig. 3d) or 10 mM ZnCl_2_ (Supplementary Fig. 4b) caused flickering as observed in a calcium-free solution (Fig. 3a and b).

Conductance measurements were performed in the presence of the chelating agent EGTA. After the overnight incubation of the protein samples with 10 mM of EGTA, we observed in 1 M KCl, 10 mM Mes, and 10 mM EGTA at pH 6.0 a conductance of 2.4 ± 0.1 nS and, at negative potential, a conductance of 2.5 ± 0.1 nS (Supplementary Fig. 4c). As with native protein, noisy traces were observed with upward spikes seen at negative voltages. In a control measurement, we removed the EGTA by the addition of 10 mM CaCl_2_ in 1 M KCl buffer and observed the previous CaCl_2_ behavior (Supplementary Fig. 4d).

### Ion selectivity

The ion selectivity was obtained from zero-current membrane potential measurements in the presence of up to 8-fold KCl concentration ratio (0.1 M KCl *versus* 0.8 M KCl). Approximately, 300–500 protein channels were reconstituted under a voltage of + 20 mV. After the saturation of channel insertion, we measured the zero-current membrane potential under a wide range of concentration gradient ratios ranging from 1.5 to 7.5. At a concentration gradient of 500 mM (0.1 M *versus* 0.5 M), the zero-current membrane potential was 28 ± 4 mV at the diluted side, revealing the preference for cations. Goldman–Hodgkin–Katz equation [27] determines the ratio of cation to anion (*P*_K__+_/*P*_Cl-_) to be 7 ± 2 for nMOMP and within the error identical for rMOMP (6 ± 1). Similarly, measurements were performed in the presence of 8-fold calcium chloride (0.1 M CaCl_2_ *versus* 0.8 M CaCl_2_) and no KCl. The zero-current membrane potential was measured as − 19 ± 1 mV, which implies that the cationic selectivity exhibited by the channel in monovalent cation (KCl) turns into anionic in the presence of divalent cation (CaCl_2_). This is consistent with the structure that shows that the narrow region of the constriction zone is lined with five negatively charged amino acid residues, which would favor the transport of cationic molecules over anionic ones. However, in the presence of calcium chloride, Ca^2 +^ binds to the negatively charged residues reversing the ion selectivity (Supplementary Fig. 3g).

### Interaction of ciprofloxacin with MOMP

**Fig. 4:**
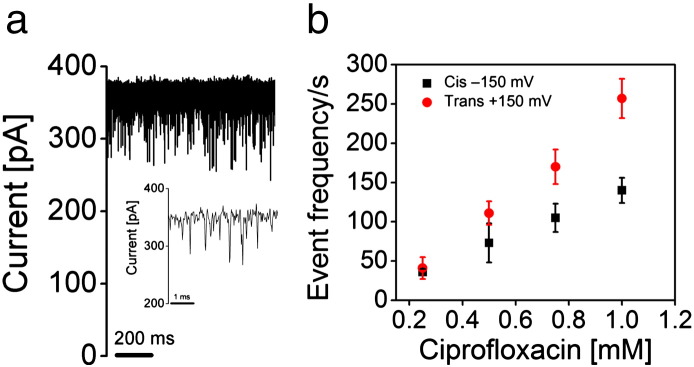
Ciprofloxacin binds to and blocks channel conductance. (a) Transient blockages are observed in the ion-current trace of single trimeric rMOMP channel in the presence of 1 M KCl, 10 mM Mes, and 10 mM CaCl_2_ (pH 6.0) when 0.5 mM ciprofloxacin is added to *trans* side. The applied transmembrane potential was + 150 mV. (b) The number of binding events increases with the increase in the concentration of the ciprofloxacin from 0.25 to 1 mM measured at 150 mV.

A single trimeric channel of MOMP was reconstituted into artificial lipid bilayers containing 1 M KCl (without CaCl_2_). Addition of 1 mM ciprofloxacin to the *trans* side of the lipid bilayer decreased the conductance by 0.2 nS to 2.2 nS, along with an increase in the current noise (Supplementary Fig. 5a and b). The stronger flickering does not allow individual blocking events to be distinguished (Supplementary Fig. 5a). Increasing the concentration of ciprofloxacin to 2 mM reduces the conductance to ~ 2.1 nS at + 150 mV (Supplementary Fig. 5a and c). In the presence 1 M KCl, 10 mM Mes, and 10 mM CaCl_2_ addition of 0.5 mM ciprofloxacin to trimeric MOMP resulted in distinct blockages, which were concentration dependent (Fig. 4a and b). The blockage events were fitted into a kinetic binding model with an on rate, *k*_on_, of ʋ/3[c] (ʋ = number of blockages per second; [c] is concentration) and an off rate, *k*_off_, of 1/τ (τ = dwell time) [28]. The average dwell time did not depend on the concentration of the ciprofloxacin and was found to be in the range of 60 μs for the voltages applied between 125 and 199 mV (Supplementary Fig. 5d and e). The values of *k*_on_ and *k*_off_ obtained are shown in Table 2. Twofold increase in the blocking events on the addition of ciprofloxacin on the *trans* side when compared to the addition to the *cis* side was observed. This suggests that ciprofloxacin accessed the binding site more readily from the *trans* side. Addition of ciprofloxacin in the presence of magnesium chloride resulted in fast flickering events (Supplementary Fig. 6), consistent with a specific modulation in function by Ca^2 +^. In fact, in the presence of CaCl_2_, we saw mainly a trimeric protein, suggesting that the MOMP trimer was itself stabilized by CaCl_2_. The interaction of ciprofloxacin with MOMP trimers in the presence of calcium chloride resulted in clean blockage events. With monomeric protein, the blocking events were replaced with flickering.

### In silico modeling

**Fig. 5:**
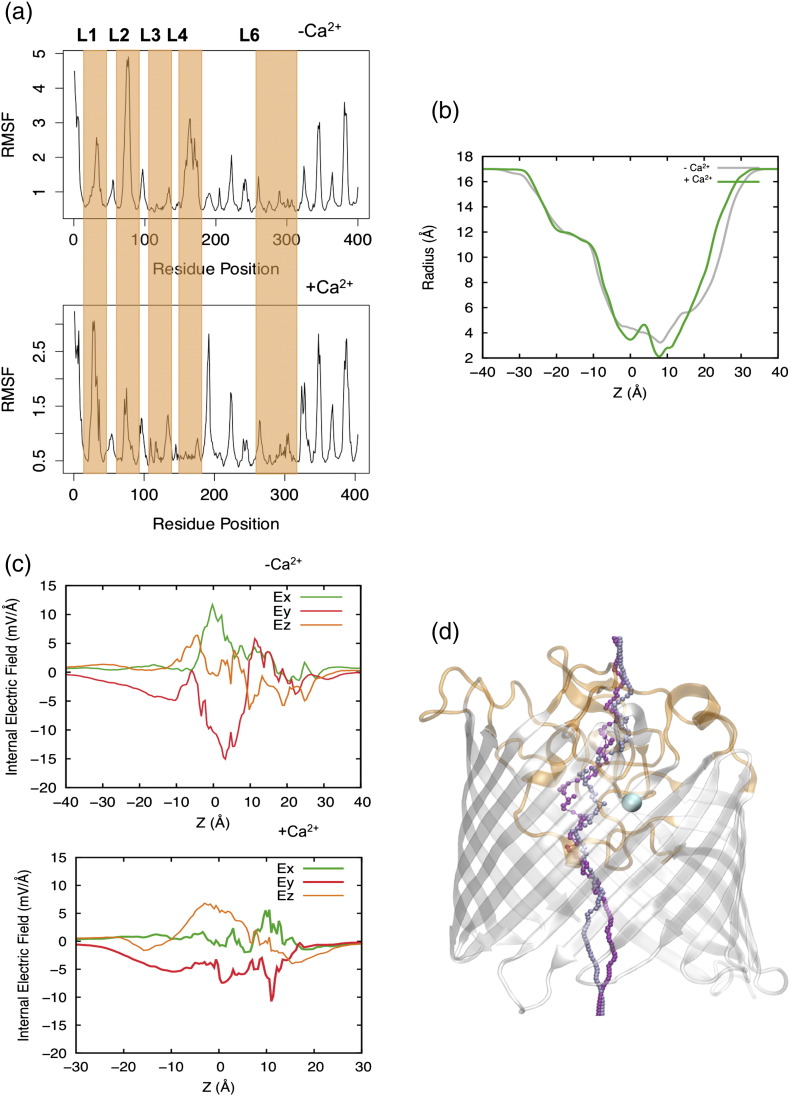

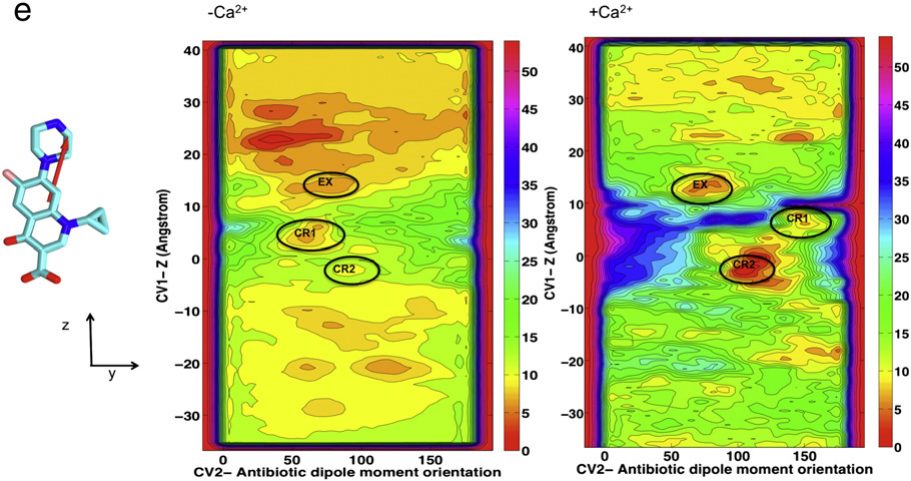
MOMP permeability. (a) RMSF of protein backbone for the two simulated structures: not Ca^2 +^ bound and Ca^2 +^ bound. The loops presenting bigger changes in fluctuation values are highlighted in orange. (b) The pore radius of MOMP monomer moving from the extracellular side to the periplasm without bound calcium (gray) and with bound calcium (green). (c) The macroscopic intrinsic electric field of MOMP with and without calcium. A reduction of the transversal components from ~ 18 mV/Å in the structure without calcium to ~ 7 mV/Å when calcium is present. (d) The putative pathway for water diffusion shifts toward the calcium ion when it is present (ice blue), while in the structure without calcium ion (purple), it avoids the cluster of negative residues that coordinate Ca^2 +^. MOMP monomer is shown in cartoon representation. (e) Free energy surface for the translocation of ciprofloxacin through MOMP without and with calcium. Antibiotic dipole moment orientation is depicted onto a licorice representation of ciprofloxacin in red. Each isocontour corresponds to a free energy difference of 2 kcal mol^− 1^. Free energy values were rescaled for each surface in order to have the absolute minimum equal to zero. The most relevant minima for ciprofloxacin translocation have been labeled for both scenarios: one in the extracellular region (EX) and two inside the constriction region (CR1, CR2).

Two rMOMP structures, one with calcium and one without, were equilibrated in silico, and the RMSD and root mean square fluctuations (RMSF) of the protein's backbone were calculated. With calcium, the structure is considerably more stable (RMSD of 1.45 Å *versus* 1.75 Å and RMSF halved). The instability was focused on the loops involved in calcium coordination (Fig. 5a). Although the empty site was occupied transiently by one of the sodium ions used to neutralize the system, its presence was not sufficient for the stabilization of the structure. Figure 5b shows that the pore size slightly increased in the absence of Ca^2 +^.

In the absence of the calcium ion (Fig. 5c), the transverse internal electric field [29] of ~ 18 mV/Å was comparable to OmpC (~ 20 mV/Å) but lower than OmpF (36 mV/Å). When calcium was present, the internal electric field was reduced to almost a third of its value due to charge screening, which in turn reduced the transversal electric field in the constriction region to ~ 7 mV/Å. The divalent ion induced a rotation of the transversal electric field and a change in the conformation of the internal loops, which led to a shift in the putative path that water was predicted to diffuse along. When calcium was not present, water avoided passing near the cluster of negative residues that bind the divalent ion located in the region (Fig. 5d), whereas when calcium was present and the transversal electric field rotated, the water pathway was shifted toward the ion position.

In order to understand the effects of the presence of the cation in the permeation of polar antibiotics, the free energy profiles were calculated for ciprofloxacin translocation for both calcium-bound and calcium-free protein. The free energy maps show that the main barrier to translocation was, as expected, located at the constriction zone (Fig. 5e). Both free energy maps share common features but differed at the calcium-binding site. In the absence of calcium, ciprofloxacin approaches the protein constriction region by entering with its positive group, which is attracted toward the negatively charged calcium-binding site (Supplementary Fig. 7). This is similar to what happens in OmpF from *E. coli*, where the negative patch on L3 modulated the translocation of antibiotics and traps a divalent cation [30].

In the presence of calcium ion (Supplementary Fig. 7), ciprofloxacin entered with the opposite orientation, pointing its carboxylic group toward the ion. Translocation of ciprofloxacin starting from the “*cis*” side required us to apply a voltage above + 100 mV to achieve this favorable orientation. Critically, molecular dynamics (MD) predicts that the antibiotic follows a different path and has a higher barrier to translocation in the presence of bound Ca^2 +^ ion.

## Discussion

We detected no difference in structure or function between the MOMP purified from *C. jejuni* and the MOMP heterologously expressed in *E. coli*. Unusually for an 18-stranded β-barrel, MOMP adopts the same trimeric arrangement as seen for OmpF and OmpC, which are 16-stranded β-barrels. Similar to these trimers, a loop from one monomer reaches across and partly into the central pore of another monomer. Whether the trimer has functional significance, other than stability, is unclear. A distinctive structural feature of MOMP is the α-helix at the N terminus. The other known outer membrane proteins with an N-terminal helix are OprB from *Pseudomonas aeruginosa* and the palmitoyl transferase PagP from *E. coli*; both proteins are monomers [31], [32]. The trimeric sucrose-specific porin (ScrY) from *Salmonella typhimurium* comprises a coiled-coil domain at the N-terminal periplasmic domain [33]. Although the secondary structure of the N terminus was not determined from its crystal structure [33], spectroscopic and biophysical analyses suggested that it forms α-helical coiled-coil complexes that might be involved in the supramolecular stabilization and low-affinity sugar binding of ScrY [34]. The role of the N-terminal α-helix in MOMP has not been experimentally investigated, but structural analysis suggests that it favors trimerization. The most striking difference between MOMP and OmpF/OmpC from *E. coli* is the presence of a Ca^2 +^-binding site in the constriction zone of MOMP. The site clearly has a high affinity for calcium, since the overexpressed protein had selectively bound the ion from the culture and held onto it during purification. The ion is bound to a number of key loops that form the constriction zone, suggesting its structural role. Addition of EDTA to isolated protein, which would remove any calcium, has been previously shown to destabilize the protein [20]. We see that EGTA addition results in noisy conductance (Supplementary Fig. 4) comparable to native protein, whereas the addition of 10 mM CaCl_2_ containing 1 M KCl gives stable conductance traces (Fig. 3c). Addition of Mg^2 +^ (Fig. 3d) or Zn^2 +^ (Supplementary Fig. 4b) did not result in a stable ion-current, suggesting that this is a specific feature of Ca^2 +^. MD analysis showed that in the absence of calcium, the loops become unstable and disrupted the order of the constriction zone. We suggest that the binding of Ca^2 +^ anchors the loops, thus ordering the eyelet, preventing them from “flopping” into the pore, and hence eliminating the spikes seen in ion-current in the absence of calcium (Fig. 3a and b).

Comparison of MOMP sequences (one each) from *C. jejuni*, *Campylobacter coli*, *Campylobacter fetus*, *Campylobacter lari*, and *Campylobacter upsaliensis* shows that three (Asp116, Glu288, and Asp289) of the five amino acids involved in metal binding are absolutely conserved. Asp120, which coordinates the metal ion in *C. jejuni* is conserved in *C. coli* and *C. upsaliensis*, but not in *C. fetus* or *C. lari* where there is a deletion of 4 aa. Gln152, which coordinates the metal ion with its side-chain oxygen, is fully conserved in *C. jejuni*, *C. coli*, and *C. upsaliensis* but is replaced by an asparagine in *C. fetus* and an aspartic acid in *C. lari* (Supplementary Fig. 2)*.* MOMP, in the absence of Ca^2 +^, is a cation-selective intermediate between OmpC (most) and OmpF (least). The calculated radius at the narrowest point in the constriction zone of MOMP is 2.6 Å, somewhat larger than OmpC (2.2 Å) and smaller than OmpF (3.3 Å). Liposome swelling studies [35] have shown that MOMP is capable of transporting larger neutral molecules (such as arabinose, glucose, mannose, α-ketoglutarate, etc.) than OmpC, consistent with a pore that is larger than that of OmpC.

The single-channel conductance for rMOMP and nMOMP from the 85H strain of *C. jejuni* was 0.7 ± 0.2 for the monomeric and 2.2 ± 0.2 nS for the trimeric state in 1 M KCl. Previously, Dé and colleagues reported lower conductances for the monomer (0.51 ± 0.04 nS) and trimer (1.5 ± 0.06 nS) [36]. Given that the different buffer condition (here at pH 6.0, rather than pH 7.4, as reported by Dé *et al.*) may reflect differences in experimental conditions, both the rMOMP and nMOMP channels from *C. jejuni* exhibit two distinct channel conductance values related to the trimer and the monomer. We are unable to determine if the monomer species represents a trimer with two closed channels or a genuine monomer arising from dissociation of the trimer. The trimer exhibits a large single-channel conductance of 2.2 nS, similar to the conductance of cyanobacterium species *Synechocystis* sp. PCC 6714 [37] and comparable to the *E. coli* OmpC (2.5 nS) [38]. The conductance of the monomer is similar to the unitary conductance of cation-selective monomeric channel *P. aeruginosa* OccD3 (around 0.7 nS) [39] and *E. coli* OmpF (1 nS; all in 1 M KCl) [40], [41]. The monomeric value is also comparable to the triton/EDTA-solubilized protein of *C. jejuni* UA580 strain [42] and *C. coli* UA30 [35], whose single-channel conductances are 0.82 and 0.53 nS, respectively. In KCl solution, the channel was selective for cations, but after the addition of CaCl_2_, the channel switched its selectivity for anions. This inversion of selectivity mirrors that seen for OmpF in the presence of MgCl_2_ [30].

The presence of Ca^2 +^ (but once again not Mg^2 +^) also altered the behavior of MOMP with ciprofloxacin in single-channel experiments (Fig. 4). The antibiotic blocked the channel with frequent binding events of short residence time (60 μs). When Ca^2 +^ ions were not present, ciprofloxacin reduced the current flow in a concentration-dependent manner (Supplementary Fig. 5a), but individual blocking events were not seen. We conclude that in the absence of the Ca^2 +^, antibiotic translocation occurs too rapidly to see individual events under our experimental conditions. Metal-ion-dependent blockages were observed with imipenem added to OmpPst1 channel from *Providencia stuartii* [28] in the presence of La^3 +^ and with enrofloxacin added to OmpF channel in the presence of Mg^2 +^ [30]. In both these cases, the ion does not co-purify with the protein or seem to have any role in stability.

The presence of a calcium ion at the constriction zone partly neutralizes the negative charges and thus in turn profoundly weakens the transverse electric field at the eyelet. The transverse field is known to play a key role in the translocation of polar molecules [43]. MD shows that the presence of the Ca^2 +^ increased the barrier to transport compared to an artificial model without the ion; a prediction entirely consistent with the experimental single-channel data. A review of the antibiotic sensitivity of *C. jejuni* showed that the organism is less susceptible to singly or doubly anionic antibiotics and to larger, dipolar ionic molecules [35]. The increased resistance to the charged antibiotics is consistent with a reduced transverse field, which would increase the barrier to translocation.

Food poisoning by ingestion of *Campylobacter* is common and, as resistant strains have emerged, is causing concern. Unlike *E. coli*, *Campylobacter* have evolved to possess only one MOMP; thus, it cannot switch between OmpC and OmpF expression as *E. coli* does to alter antibiotic entry to the cell. In structural terms, MOMP is distinct from both OmpC and OmpF; however, in terms of pore size, it is intermediate. Uniquely, MOMP may rely on a permanently bound Ca^2 +^ ion to modulate translocation across the outer membrane.

## Materials and Methods

### Molecular biology

A gene encoding MOMP from *C. jejuni* strain 85H (Uniprot entry number: Q659I5) was synthesized with codon optimization for *E. coli* by Eurofins MWG (Germany). The gene carried an NcoI enzymatic cleavage site at the 5′-end and a stop codon followed by HindIII site at the 3′-end. The *momp* gene was cloned into a pTAMAHisTEV expression vector. The pTAMAHisTEV vector was created by placing a TamA signal peptide into the T7 promoter ampicillin resistance pHisTEV vector [44]. Consequently, the pTAMAHisTEV plasmid expressed a protein with a TAMA signal peptide at the N terminus, followed by a histidine tag, then the TEV recognition sequence ENLYFQG, and finally the N-terminus of the target protein (here MOMP).

### Protein expression

The plasmid pTAMAHisTEV harboring the MOMP gene was transformed into C43(DE3) competent cells. A 250-ml LB startup culture containing 100 μg ml^− 1^ ampicillin was incubated at 37 °C and at 200 RPM. Then, 20 ml of startup culture was transferred to 10 × 1 L LB containing the same amount of ampicillin and was grown at 37 °C until an OD_600_ of ~ 0.6. At this stage, cells were induced with 0.4 mM IPTG, the temperature was dropped to 25 °C, and growth continued for 16 h. Cells were harvested by centrifugation at 6200*g* (JLA8.1000 rotor, Beckman Coulter).

Cell pellets were resuspended in lysis buffer containing 20 mM Tris–HCl (pH 8.0), 300 mM NaCl, 10% glycerol, 20 μg ml^− 1^ DNAse, 100 μg ml^− 1^ lysozyme (both Sigma-Aldrich), and EDTA-free protease inhibitor cocktail (Roche). Cells were lysed by two passes through a chilled cell disruptor at 30 kpsi. Cellular debris was removed by centrifugation at 10,000*g* (JA 25.50 rotor, Beckman Coulter). The membrane fraction was retained by ultracentrifugation at 100,000*g* (50.2 Ti rotor, Beckman Coulter) at 4 °C for 1 h. A two-step process failed in our hands, in which the inner membrane was with *N*-lauroyl sarcosine and then discarded, followed by solubilization of the outer membrane with SB3.14, N,N-dimethyldodecylamine N-oxide (LDAO), *n*-octylpolyoxyethylene (Octyl-POE), or other detergents; the MOMP remained insoluble. Direct solubilization of total cell membrane with either 1% SB3.14 or 5% Elugent™ gave much improved extraction. As TEV protease was inactive in the presence of SB3.14, extraction was carried out in 20 mM Tris–HCl (pH 8.0), 150 mM NaCl, 5% (vol/vol) Elugent™ (Merck Millipore), and EDTA-free protease inhibitor cocktail using a tissue grinder and subsequent incubation at 4 °C overnight with gentle rotation. Insoluble material was removed by centrifugation (100,000*g* for 1 h). Elugent concentration was reduced by dilution to a final concentration of 1.25% (vol/vol). This protein solution was cycled over Ni-NTA column at 4 °C overnight using a peristaltic pump to ensure binding. The column was washed extensively with a buffer containing 20 mM Tris–HCl (pH 8.0), 30 mM imidazole, 150 mM NaCl, and 0.25% (vol/vol) Elugent™. MOMP was eluted with 2 column volume (CV) of buffer containing 250 mM imidazole. The eluent was supplemented with 1 mg His_6_-tagged TEV protease and dialyzed in SnakeSkin tubing (Thermo Scientific) against a buffer containing 10 mM imidazole at room temperature overnight. The dialyzed sample was passed through a 0.45-μm syringe filter to remove insoluble particles before the sample was applied to 2-ml Ni-NTA resin. Cleaved MOMP was collected from the flow-through fraction. MOMP was further purified by gel filtration (16/60 Superdex 200 pg; GE Healthcare) on an Äkta Express purifier during which Elugent™ was exchanged for 0.45% (wt/vol) C_8_E_4_. MOMP was concentrated to 10 mg ml^− 1^ for crystallization. Purity and integrity were monitored on SDS-PAGE (NuPAGE, Invitrogen) and mass spectrometry. SeMet-labeled MOMP was produced according to the metabolic inhibition method [45] using SeMet media from Molecular Dimensions and was purified as described above.

*C. jejuni* 85H strain [46] was grown according to Bolla *et al.* [11]. *C. jejuni* strain was spread onto a blood agar plate and incubated for 24 h at 42 °C. Bacteria were recovered with 1 ml of 2YT medium. Four Columbia agar plates supplemented with the appropriate amount of *Campylobacter* selective antibiotics supplement (Oxoid) were inoculated with 150 μl of the recovered bacteria solution and incubated for 48 h at 42 °C. 2YT medium was used to recover the bacteria, which were subsequently inoculated on 40 plates of Columbia agar and incubated for 48 h at 42 °C. Subsequently, each plate was rinsed with 5 ml of 10 mM Tris–EDTA buffer (pH 7.4) and agitated for 15 min at room temperature; then, bacterial suspension was recovered and the OD_600_ checked. From this point, all the following steps were performed at 4 °C. The bacteria were pelleted by centrifugation at 10,000*g* for 30 min. The pellet was resuspended in 200 ml of 200 mM Glycine–HCl (pH 2.2) and agitated for 15 min. Bacteria were harvested by centrifugation at 10,000*g* for 30 min and washed in 100 mM Tris–HCl (pH 7.4). Cells resuspended in Tris–HCl 10 mM (pH 7.4) were then lysed by using two passes at 30 Kpsi through a high-pressure cell disruption for micro volumes (Constant System Ltd). Unbroken cells were removed by spinning the cell lysate at 10,000*g* for 30 min. The supernatant was kept and spun down at 100,000*g* for 1 h. The pellet was then homogenized in 10 mM Tris–HCl (pH 7.4) and 0.1% (wt/vol) of sodium lauryl sarcosinate (Sigma) and was left rocking for 30 min. The outer membrane was recovered by ultracentrifugation at 100,000*g* for 1 h. The supernatant containing the inner membrane protein fraction was discarded, and the pellet was homogenized with 20 mM sodium phosphate buffer (pH 7.4) and 1% of Octyl-POE (Bachem AG) and was left rocking at 4 °C for 30 min. Solubilized proteins were recovered by ultracentrifugation at 100,000*g* for 1 h. Protein was loaded onto a MonoQ HR ion exchange column (GE Healthcare) and was equilibrated with 5 CV of buffer A (30 mM Na_2_HPO_4_, 10 mM NaCl, and 0.6% Octyl-POE). The bounded proteins were eluted stepwise with 5, 12, 20, 70, and 100% of buffer A supplemented with 1 M NaCl. The chromatography was performed on ÄKTA Explorer 10 system (GE Healthcare). Each fraction was examined with SDS-PAGE and Western blot with specific antibodies. Eluted fractions containing MOMP were collected and concentrated to 5 ml and injected onto a Superdex 200 16/60 GL (GE Healthcare) column equilibrated with 2 CV of 20 mM Tris–HCl (pH 8.0), 150 mM NaCl, and 0.45% (wt/vol) C_8_E_4_. Fractions containing MOMP were combined and concentrated to 10 mg ml^− 1^.

### Crystallization

Recombinantly expressed MOMP crystallized in 50 mM sodium citrate (pH 4.25), 35% (vol/vol) PEG 400, and 70 mM KCl in a hanging-drop vapor-diffusion experiment at 20 °C with 500 μl reservoir solution and a crystallization droplet of 2 μl protein and 1 μl precipitant. SeMet MOMP crystallized under identical conditions. Crystals grew to full size after 1 week and were hexagonal in shape.

MOMP purified from *Campylobacter* crystallized in 0.05 M calcium chloride, 0.05 M barium chloride, 0.1 M Tris (pH 7.5), and 30% (vol/vol) PEG 400. Crystals were optimized using hanging-drop vapor-diffusion technique. Crystals appeared after 3 days in a drop made of 1 μl protein and 1 μl reservoir made of 0.05 M calcium chloride, 0.05 M barium chloride, 0.1 M Tris (pH 8), and 32% (vol/vol) PEG 400. In all cases, crystals were flash cooled before data collection, but no cryoprotectant was added.

### Data collection, and structure determination and refinement

X-ray data of recombinant SeMet crystals were collected at beamline ID23-1 in ESRF, Grenoble, France and native data at Diamond. Data were processed with Xia2 [47]. Both belong to spacegroup P6_3_ with one monomer per asymmetric unit. The SeMet structure was solved by single-wavelength anomalous dispersion to a 2.7-Å resolution with Autosolve program of Phenix [48]. Using this as search model, the 2.1-Å native structure was solved with the PHASER MR from Phenix [49]. The model was built using Arp/Warp [50]. X-ray data from a crystal of MOMP purified from *Campylobacter* crystals were collected in house using a Rigaku Micromax™-007HF Cu anode with VariMax optics and a Rigaku Saturn 944 + CCD detector and processed with Xia2 [47]. The structure was solved to a resolution of 2.9 Å by molecular replacement using the recombinant MOMP structure as a search model with the program Phaser [49]. Crystals belong to spacegroup P2_1_2_1_2_1_ with three molecules per asymmetric unit and a Matthew's coefficient of 45%.

The three models were manually completed in Coot [51] and refined with REFMAC [52]. Structures were validated with Molprobity [53]. Data collection and refinement statistic are listed in Table 1. The webserver “CheckmyMetal” validates the identification as calcium [54]. Additionally, the difference electron density map showed a residual elongated density that was modeled as an ethylene glycol like the molecule (from PEG 400 or C8E4) in the recombinant MOMP. This molecule sits below the constriction zone underneath loop 3.

### Single-channel conductance measurements

A planar lipid bilayer was formed using solvent-free lipid bilayer technique [55]. In brief, the cuvettes used for our bilayer experiments consist of *cis* and *trans* chambers separated by a 25-μm thick teflon film (Goodfellow) carrying an aperture with a diameter of 40–70 μm. The spherical hole in the teflon film was made by a high-voltage cathode discharge (Electrotechnic Products). In order to form the lipid bilayer, the aperture is pre-painted with 1 μl of 1% hexadecane in hexane. Due to its high mechanical and chemical stability, 5% solution of diphytanoyl phosphatidylcholine (DPhPC, Avanti Polar Lipids) is commonly used [56]. The bilayer it forms allows for the insertion of transmembrane pores. The chambers are filled with electrolyte solution, which usually consist of 1 M KCl and 10 mM Mes (pH 6.0) with a total solution volume of 2.5 ml. MOMP was added to the *cis* side (which is the electrical ground or reference potential) of the chamber at a final concentration of 2 ng ml^− 1^, and the channel insertion was facilitated by the rapid mixing of the contents of the chamber while applying a transmembrane potential of − 199 mV. Electrical recordings were made through a pair of Ag/AgCl electrodes (World Precision Instruments), attached to an Axon Instruments 200B amplifier (Axon Instruments Inc.) in the voltage clamp mode. Data were filtered by a low-pass Bessel filter at 10 kHz and directly saved into the computer memory with a sampling frequency of 50 kHz. Data analyses were performed using Clampfit 10.0 software (Axon Instruments Inc). As observed previously [35], multiple channel events for MOMP were non-homogeneous and hence were not analyzed further (Supplementary Fig. 3h).

### Ion-selectivity measurements

The ion selectivity of ~ 44-kDa MOMP was carried out by measuring the zero-current membrane potential as described elsewhere [27]. The Teflon cuvettes consist of two chambers, with a pre-designed spherical hole having a diameter of 0.5–0.5 mm^2^. In chloroform, 2% DPhPC serves as the pre-painting solution, and 1% solution of DPhPC in *n*-decane serves as the lipid for the formation of black lipid membranes. After the formation of a stable membrane, 50–100 ng/ml of MOMP protein was added to both sides of the Teflon chambers containing 0.1 M KCl solution. After the incorporation of 200–500 channels, a salt gradient was established by adding 3 M solution of KCl on one side of the membrane, and an equal volume of 0.1 M solution of KCl was added on the other side with uniform stirring of the contents. The resulting zero-current membrane potentials were measured using the high-impedance electrometer (Keithley 617).

### MD

The high-resolution X-ray structure of trimeric MOMP was used as starting coordinates for MD simulations. Loop 2 from Ala77 to Glu81 was missing in the crystal structure and was modeled using MODELLER [57] that considered the optimal structure among 100 generated models. Amino acid residues were simulated in its ionization state at neutral pH. The entire trimer was embedded in a pre-equilibrated 1-palmitoyl-2-oleoyl-sn-glycero-3-phosphocholine bilayer of 471 lipids and the system was oriented in order to center the protein at the origin of the coordinate system and align the channel along the *z*-axis (positive z: extracellular side; negative z: periplasmic side). We added 53 sodium ions to neutralize the system total charge. The system was solvated with ~ 40,997 TIP3P water molecules (simulation box size: 13.72, 23.80, 10.71 nm; total number of atoms: ~ 204 k). Simulations were performed with and without the calcium ion to evaluate its effect on the structure and dynamics of loops.

After 1 ps of energy minimization (conjugate gradients), a slow heating from 10 to 300 K was carried out for 1 ns. During this stage, positional restraints were applied on the protein α-carbons (all three dimensions) and on the lipids' phosphorus atoms (along z only). After releasing the constraints on the 1-palmitoyl-2-oleoyl-sn-glycero-3-phosphocholine, an equilibration stage follows for 4 ns in the isothermal-isobaric (NPT) ensemble at 1.0 bar and 300 K. Finally, 0.9-μs MD simulations were performed in the canonical (NVT) ensemble after the elimination of the protein restraints.

The NPT equilibration was performed with the program NAMD [58], with 1.0 fs time step, and by treating long-range electrostatics with the soft particle mesh Ewald method (64 grid points and order 4 with direct cutoff at 1.0 nm and 1.0 Å grid size). Pressure control was applied using the Nose–Hoover method (extended Lagrangian) with isotropic cell, integrated with the Langevin Dynamics (200 fs and 100 fs of piston period and decay, respectively). The latter was also applied for temperature control with 200-fs thermostat damping time. Production run in the NVT ensemble was performed with the ACEMD code [59] compiled for graphic processing units (GPUs) by rescaling the hydrogen mass to 4 au and increasing the time step up to 4.0 fs [60]. The Langevin thermostat was used with 1-ps damping time. Soft particle mesh Ewald was used to treat the electrostatics as for the equilibration stage. The Amber99SB-ILDN force field [61] was used for the protein and lipids, and the TIP3P [62] for waters.

The GAFF force field parameters [63] were used to describe ciprofloxacin (DrugBank [64] n. DB00537). Partial atomic charges were evaluated according to the restrained electrostatic potential (RESP) approach [65]: the molecule was first optimized at the HF/6-31G(d) level, up to a convergence in energy of 10^− 5^ au, using the Gaussian03 package [66]. Atomic RESP charges were derived from the electrostatic potential using the antechamber module of the AMBER package [67]. Parameters are freely available† [68].

Starting from the final configuration of the MOMP simulation without the calcium ion described above, the antibiotic was placed inside the lumen of the first monomer. The difference between the *z*-coordinate of the center of mass (com) of the antibiotic two-ring system and the *z*-coordinate of the com of the protein monomer was + 32.9 Å. A thousand steps of energy minimization were performed. The equilibration stage followed for 1 ns in the NVT ensemble at 300 K as described hereinbefore. Well-tempered metadynamics simulation (500 ns) was performed with the ACEMD code, until the first effective translocation through the protein constriction region was observed [69], [70]. Then, four configurations were randomly selected, two with the antibiotic located in the extracellular vestibule and two in the periplasmic vestibule. Correspondingly, four multiple walkers [71] were set to extend the metadynamics reconstruction of the free energy surface. Two biased collective variables were used, namely, the antibiotic position and the projection of the dipole moment of the antibiotic onto the *x*-axis of the channel. In practice, the “position” Δ*z* was defined as the difference of the *z*-coordinate between the com of the antibiotic two-ring system and that of the porin first monomer. We run 4 × 1 μs, arriving to a total simulation time of 4.5 μs. During the metadynamics, energy biases were added every 1.25 ps to each collective variable (initial height: 1.0 kcal mol^− 1^; σ 0.3 kcal mol^− 1^ and 5.0 degree for position and dipole moment orientation, respectively). Well-tempered Δ*T* was 5000 K.

### Accession numbers

Coordinates and structure factors have been deposited in the Protein Data Bank with the ID codes 5ldt and 5ldv.

## Acknowledgments

L.G.M.F. and S.A.-G. are funded by EU FP7-PEOPLE-2013-ITN Translocation network Nr. 607694. The research leading to these results was conducted as part of the Translocation consortium (www.translocation.com) and has received support from the Innovative Medicines Initiatives Joint Undertaking under Grant Agreement no.115525, resources of which are composed of financial contribution from the European Union's seventh framework programme (FP7/2007-2013) and of the EFPIA companies in kind contribution. J.H.N. is Royal Society Wolfson Merit Award holder, Senior Investigator Wellcome Trust (WT100209MA), and Chinese Academy of Science 1000 Talent Scholar. M.C. thanks the PRACE consortium for the use of the Research Infrastructure CURIE based in France at TGCC through the project Tier-0 nr. RA2699. The use of beamlines at both Diamond and ESRF is acknowledged.

## Figures and Tables

**Table 1:** Data collection, refinement, and validation statistic

	MOMP Native	MOMP Recombinant	Recombinant MOMP^SeMet^
Space group	P2_1_2_1_2_1_	P6_3_	P6_3_
Cell dimensions *a*, *b*, *c* (Å)	94.4, 99.4172.2	90.6, 90.6, 104.4	94.3, 94.3, 102.0
Cell dimensions α, β, γ (°)	90, 90, 90	90, 90, 120	90, 90, 120
Resolution (Å)	47.75–2.9(3.06–2.9)	39.2–2.1(2.17–2.1)	2.82
*R*_merge_	0.14 (0.86)	0.06 (0.72)	0.076 (0.90)
Completeness	99.9 (99.8)	99.5 (99.5)	99.7 (99.7)
Multiplicity	7.3 (7.2)	13.5 (13.6)	14.6 (15.0)
*I*/σ(*I*)	12.9 (3.0)	29.89 (3.92)	23.1 (4.3)

*Refinement*			
*R*_factor_/*R*_free_ (%)	23.9/27.1	19/22.27	
No. of unique reflections	36,693	28,313	
No. of residues	405	403	
Water	5	127	
Bonds length (Å)	0.01	0.01	
Bonds angles (°)	1.9	1.41	
MolProbity score	1.70	1.03	

Values in parentheses are for the highest-resolution shell.

**Table 2:** Kinetics of ciprofloxacin entry into MOMP channel

Rate constants	0.5 mM ciprofloxacin
*k*_on *cis*_ (× 10^3^ M^− 1^ s^− 1^)	47 ± 10
*k*_on *trans*_ (× 10^3^ M^− 1^ s^− 1^)	73 ± 7
*k*_off_ (× 10^3^ s^− 1^)	16 ± 1

Experimental conditions: 1 M KCl, 10 mM Mes, 10 mM CaCl_2_, and 0.5 mM ciprofloxacin (pH 6.0); *T* = 20 °C. The applied transmembrane voltage was 150 mV.

## Appendix A Supplementary Data

Supplementary material 1. Image 2

Supplementary material 2. Image 3

Supplementary material 3. Image 4

## Footnotes

† www.dsf.unica.it/translocation/db
