# Supplementary material for: MOMP from Campylobacter jejuni Is a Trimer of 18-Stranded β-Barrel Monomers with a Ca2 + Ion Bound at the Constriction Zone
Source: J Mol Biol. 2016 Nov 6;428(22):4528–43. doi: 10.1016/j.jmb.2016.09.021 (PMC5090048; doi:10.1016/j.jmb.2016.09.021)
Supplement: Supplementary file 2 — Supplementary material 2. [file mmc2.pdf]

Full wwPDB X-ray Structure Validation Report (i)

Jun 28, 2016 – 02:34 PM BST

PDB ID	:	5LDV
Title	:	Crystal Structures of MOMP from Campylobacter jejuni
Authors	:	Naismith, J.H.; Ferrara, L.M.G.; Wallat, G.D.; Moynie, L.
Deposited on	:	2016-06-28
Resolution	:	2.10 Å(reported)

This is a Full wwPDB X-ray Structure Validation Report.
We welcome your comments at validation@mail.wwpdb.org
A user guide is available at
http://wwpdb.org/validation/2016/XrayValidationReportHelp
with specific help available everywhere you see the (i) symbol.

CONFIDENTIAL VALIDATION REPORT

The following versions of software and data (see references (i)) were used in the production of this report:

MolProbity	:	4.02b-467
Mogul	:	1.7.1 (RC1), CSD as537be (2016)
Xtriage (Phenix)	:	1.9-1692
EDS	:	rb-20027790
Percentile statistics	:	20151230.v01 (using entries in the PDB archive December 30th 2015)
Refmac	:	5.8.0135
CCP4	:	6.5.0
Ideal geometry (proteins)	:	Engh & Huber (2001)
Ideal geometry (DNA, RNA)	:	Parkinson et al. (1996)
Validation Pipeline (wwPDB-VP)	:	rb-20027790

1 Overall quality at a glance (i)

The following experimental techniques were used to determine the structure:
X-RAY DIFFRACTION

The reported resolution of this entry is 2.10 Å.

Percentile scores (ranging between 0-100) for global validation metrics of the entry are shown in the following graphic. The table shows the number of entries on which the scores are based.

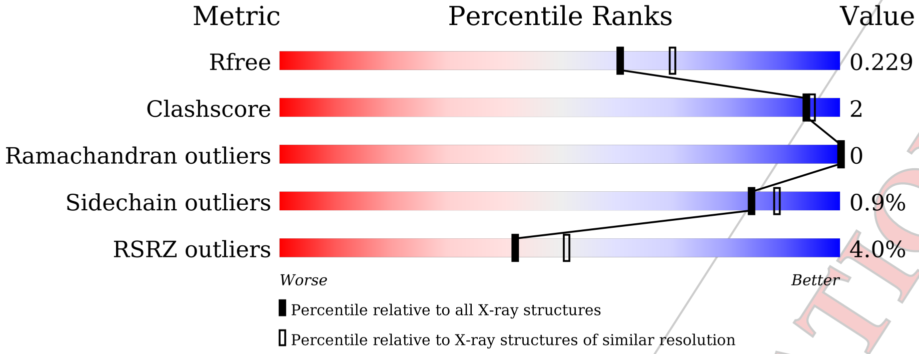

Metric	Whole archive (#Entries)	Similar resolution (#Entries, resolution range(Å))
R_{free}	91344	3939 (2.10-2.10)
Clashscore	102246	4460 (2.10-2.10)
Ramachandran outliers	100387	4413 (2.10-2.10)
Sidechain outliers	100360	4414 (2.10-2.10)
RSRZ outliers	91569	3948 (2.10-2.10)

The table below summarises the geometric issues observed across the polymeric chains and their fit to the electron density. The red, orange, yellow and green segments on the lower bar indicate the fraction of residues that contain outliers for >=3, 2, 1 and 0 types of geometric quality criteria. A grey segment represents the fraction of residues that are not modelled. The numeric value for each fraction is indicated below the corresponding segment, with a dot representing fractions <=5% The upper red bar (where present) indicates the fraction of residues that have poor fit to the electron density. The numeric value is given above the bar.

Mol	Chain	Length	Quality of chain
1	A	408	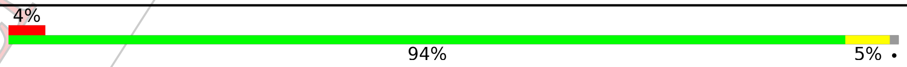

The following table lists non-polymeric compounds, carbohydrate monomers and non-standard residues in protein, DNA, RNA chains that are outliers for geometric or electron-density-fit criteria:

Mol	Type	Chain	Res	Chirality	Geometry	Clashes	Electron density
3	1PE	A	502	-	-	-	X
4	OCT	A	505	-	-	-	X

Continued on next page...

CONFIDENTIAL VALIDATION REPORT

Continued from previous page...

Mol	Type	Chain	Res	Chirality	Geometry	Clashes	Electron density
4	OCT	A	506	-	-	-	X

CONFIDENTIAL VALIDATION REPORT

2 Entry composition (i)

There are 5 unique types of molecules in this entry. The entry contains 3287 atoms, of which 0 are hydrogens and 0 are deuteriums.

In the tables below, the ZeroOcc column contains the number of atoms modelled with zero occupancy, the AltConf column contains the number of residues with at least one atom in alternate conformation and the Trace column contains the number of residues modelled with at most 2 atoms.

- Molecule 1 is a protein called MOMP porin.

Mol	Chain	Residues	Atoms					ZeroOcc	AltConf	Trace
			Total	C	N	O	S			
1	A	403	3111	1971	513	624	3	0	5	0

There are 4 discrepancies between the modelled and reference sequences:

Chain	Residue	Modelled	Actual	Comment	Reference
A	1	GLY	-	expression tag	UNP Q659I5
A	2	ALA	-	expression tag	UNP Q659I5
A	3	MET	-	expression tag	UNP Q659I5
A	4	GLY	THR	engineered mutation	UNP Q659I5

- Molecule 2 is CALCIUM ION (three-letter code: CA) (formula: Ca).

Mol	Chain	Residues	Atoms		ZeroOcc	AltConf
			Total	Ca		
2	A	1	1	1	0	0

- Molecule 3 is PENTAETHYLENE GLYCOL (three-letter code: 1PE) (formula: $C_{10}H_{22}O_6$).

CONFIDENTIAL VALIDATION REPORT

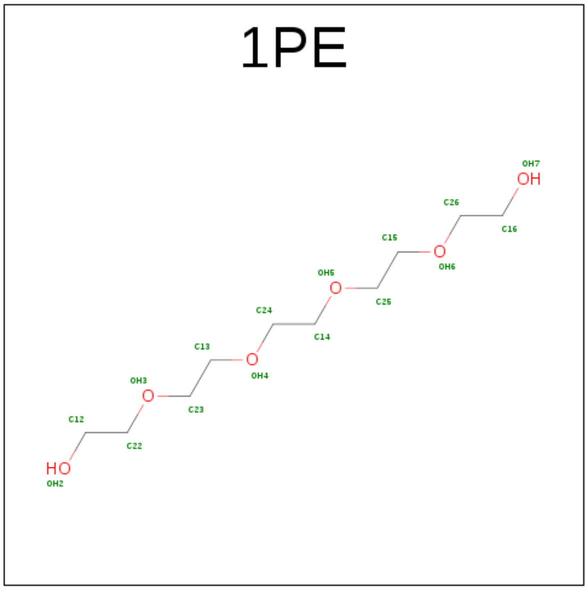

Mol	Chain	Residues	Atoms			ZeroOcc	AltConf
			Total	C	O		
3	A	1	16	10	6	0	0

- Molecule 4 is N-OCTANE (three-letter code: OCT) (formula: C_8H_{18}).

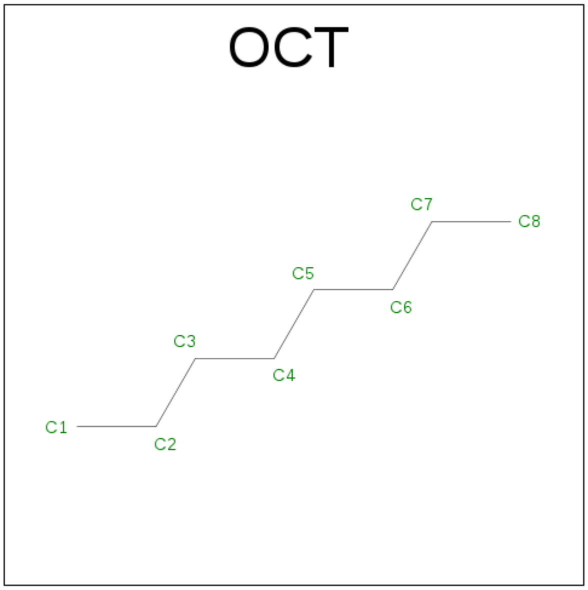

Mol	Chain	Residues	Atoms		ZeroOcc	AltConf
4	A	1	Total 8	C 8	0	0
4	A	1	Total 8	C 8	0	0
4	A	1	Total 8	C 8	0	0

Continued on next page...

CONFIDENTIAL VALIDATION REPORT

Continued from previous page...

Mol	Chain	Residues	Atoms		ZeroOcc	AltConf
			Total	C		
4	A	1	8	8	0	0

- Molecule 5 is water.

Mol	Chain	Residues	Atoms		ZeroOcc	AltConf
			Total	O		
5	A	127	127	127	0	0

CONFIDENTIAL VALIDATION REPORT

3 Residue-property plots (i)

These plots are drawn for all protein, RNA and DNA chains in the entry. The first graphic for a chain summarises the proportions of errors displayed in the second graphic. The second graphic shows the sequence view annotated by issues in geometry and electron density. Residues are color-coded according to the number of geometric quality criteria for which they contain at least one outlier: green = 0, yellow = 1, orange = 2 and red = 3 or more. A red dot above a residue indicates a poor fit to the electron density (RSRZ > 2). Stretches of 2 or more consecutive residues without any outlier are shown as a green connector. Residues present in the sample, but not in the model, are shown in grey.

- Molecule 1: MOMP porin

Chain A:

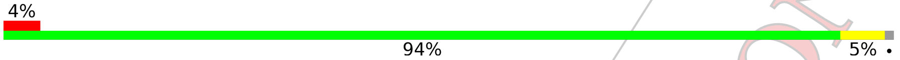

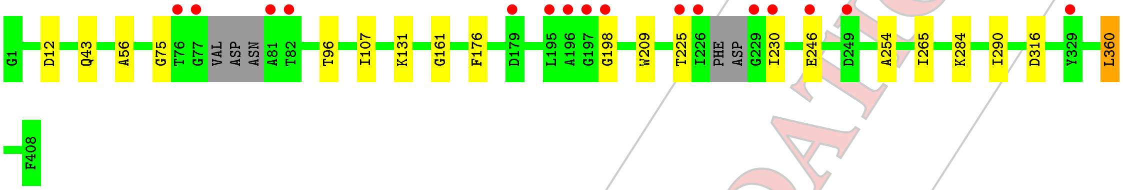

CONFIDENTIAL VALIDATION REPORT

4 Data and refinement statistics (i)

Property	Value	Source
Space group	P 63	Depositor
Cell constants<br>a, b, c, α, β, γ	90.63Å 90.63Å 104.40Å<br>90.00° 90.00° 120.00°	Depositor
Resolution (Å)	39.24 – 2.10<br>39.24 – 2.10	Depositor<br>EDS
% Data completeness<br>(in resolution range)	99.5 (39.24-2.10)<br>99.5 (39.24-2.10)	Depositor<br>EDS
R_{merge}	0.06	Depositor
R_{sym}	(Not available)	Depositor
$< I/\sigma(I) >$ [1]	3.86 (at 2.10Å)	Xtriage
Refinement program	REFMAC 5.8.0073	Depositor
R, R_{free}	0.193 , 0.222<br>0.203 , 0.229	Depositor<br>DCC
R_{free} test set	1429 reflections (5.32%)	DCC
Wilson B-factor (Å^2)	36.3	Xtriage
Anisotropy	0.036	Xtriage
Bulk solvent k_{sol}(e/Å^3), B_{sol}(Å^2)	0.35 , 51.2	EDS
L-test for twinning[2]	$< \|L\| > = 0.50$, $< L^2 > = 0.34$	Xtriage
Estimated twinning fraction	0.055 for h,-h-k,-l	Xtriage
F_o,F_c correlation	0.95	EDS
Total number of atoms	3287	wwPDB-VP
Average B, all atoms (Å^2)	42.0	wwPDB-VP

Xtriage's analysis on translational NCS is as follows: *The largest off-origin peak in the Patterson function is 5.02% of the height of the origin peak. No significant pseudotranslation is detected.*

[1] Intensities estimated from amplitudes.

[2] Theoretical values of $< |L| >$, $< L^2 >$ for acentric reflections are 0.5, 0.333 respectively for untwinned datasets, and 0.375, 0.2 for perfectly twinned datasets.

CONFIDENTIAL VALIDATION REPORT

5 Model quality (i)

5.1 Standard geometry (i)

Bond lengths and bond angles in the following residue types are not validated in this section: CA, OCT, 1PE

The Z score for a bond length (or angle) is the number of standard deviations the observed value is removed from the expected value. A bond length (or angle) with $|Z| > 5$ is considered an outlier worth inspection. RMSZ is the root-mean-square of all Z scores of the bond lengths (or angles).

Mol	Chain	Bond lengths		Bond angles	
		RMSZ	#$|Z|$ >5	RMSZ	#$|Z|$ >5
1	A	0.53	0/3179	0.71	0/4310

There are no bond length outliers.

There are no bond angle outliers.

There are no chirality outliers.

There are no planarity outliers.

5.2 Too-close contacts (i)

In the following table, the Non-H and H(model) columns list the number of non-hydrogen atoms and hydrogen atoms in the chain respectively. The H(added) column lists the number of hydrogen atoms added and optimized by MolProbity. The Clashes column lists the number of clashes within the asymmetric unit, whereas Symm-Clashes lists symmetry related clashes.

Mol	Chain	Non-H	H(model)	H(added)	Clashes	Symm-Clashes
1	A	3111	0	2970	11	0
2	A	1	0	0	0	0
3	A	16	0	22	2	0
4	A	32	0	72	0	0
5	A	127	0	0	0	0
All	All	3287	0	3064	11	0

The all-atom clashscore is defined as the number of clashes found per 1000 atoms (including hydrogen atoms). The all-atom clashscore for this structure is 2.

All (11) close contacts within the same asymmetric unit are listed below, sorted by their clash magnitude.

CONFIDENTIAL VALIDATION REPORT

Atom-1	Atom-2	Interatomic distance (Å)	Clash overlap (Å)
1:A:96:THR:HG23	1:A:107:ILE:HD13	1.89	0.54
1:A:198:GLY:HA2	1:A:225:THR:HG22	1.92	0.50
1:A:161:GLY:HA2	1:A:176:PHE:O	2.17	0.45
1:A:230:ILE:CG2	1:A:265:ILE:HD11	2.48	0.44
1:A:254:ALA:HB2	1:A:284:LYS:HB2	2.01	0.43
1:A:131:LYS:HZ3	3:A:502:1PE:H252	1.84	0.42
1:A:12:ASP:OD2	1:A:56:ALA:O	2.39	0.41
1:A:43:GLN:O	1:A:75:GLY:HA3	2.21	0.41
1:A:131:LYS:NZ	3:A:502:1PE:H252	2.36	0.40
1:A:360:LEU:C	1:A:360:LEU:HD23	2.42	0.40
1:A:209:TRP:CZ3	1:A:290[A]:ILE:HD12	2.57	0.40

There are no symmetry-related clashes.

5.3 Torsion angles (i)

5.3.1 Protein backbone (i)

In the following table, the Percentiles column shows the percent Ramachandran outliers of the chain as a percentile score with respect to all X-ray entries followed by that with respect to entries of similar resolution.

The Analysed column shows the number of residues for which the backbone conformation was analysed, and the total number of residues.

Mol	Chain	Analysed	Favoured	Allowed	Outliers	Percentiles
1	A	402/408 (98%)	391 (97%)	11 (3%)	0	100 100

There are no Ramachandran outliers to report.

5.3.2 Protein sidechains (i)

In the following table, the Percentiles column shows the percent sidechain outliers of the chain as a percentile score with respect to all X-ray entries followed by that with respect to entries of similar resolution.

The Analysed column shows the number of residues for which the sidechain conformation was analysed, and the total number of residues.

Mol	Chain	Analysed	Rotameric	Outliers	Percentiles
1	A	321/321 (100%)	318 (99%)	3 (1%)	84 89

CONFIDENTIAL VALIDATION REPORT

All (3) residues with a non-rotameric sidechain are listed below:

Mol	Chain	Res	Type
1	A	246	GLU
1	A	316	ASP
1	A	360	LEU

Some sidechains can be flipped to improve hydrogen bonding and reduce clashes. There are no such sidechains identified.

5.3.3 RNA (i)

There are no RNA molecules in this entry.

5.4 Non-standard residues in protein, DNA, RNA chains (i)

There are no non-standard protein/DNA/RNA residues in this entry.

5.5 Carbohydrates (i)

There are no carbohydrates in this entry.

5.6 Ligand geometry (i)

Of 6 ligands modelled in this entry, 1 is monoatomic - leaving 5 for Mogul analysis.

In the following table, the Counts columns list the number of bonds (or angles) for which Mogul statistics could be retrieved, the number of bonds (or angles) that are observed in the model and the number of bonds (or angles) that are defined in the chemical component dictionary. The Link column lists molecule types, if any, to which the group is linked. The Z score for a bond length (or angle) is the number of standard deviations the observed value is removed from the expected value. A bond length (or angle) with $|Z| > 2$ is considered an outlier worth inspection. RMSZ is the root-mean-square of all Z scores of the bond lengths (or angles).

Mol	Type	Chain	Res	Link	Bond lengths			Bond angles		
					Counts	RMSZ	$\#\|Z\|>2$	Counts	RMSZ	$\#\|Z\|>2$
3	1PE	A	502	-	15,15,15	0.78	0	14,14,14	0.80	0
4	OCT	A	503	-	7,7,7	0.23	0	6,6,6	0.45	0
4	OCT	A	504	-	7,7,7	0.29	0	6,6,6	0.38	0
4	OCT	A	505	-	7,7,7	0.26	0	6,6,6	0.37	0
4	OCT	A	506	-	7,7,7	0.35	0	6,6,6	0.29	0

In the following table, the Chirals column lists the number of chiral outliers, the number of chiral

CONFIDENTIAL VALIDATION REPORT

centers analysed, the number of these observed in the model and the number defined in the chemical component dictionary. Similar counts are reported in the Torsion and Rings columns. '-' means no outliers of that kind were identified.

Mol	Type	Chain	Res	Link	Chirals	Torsions	Rings
3	1PE	A	502	-	-	0/13/13/13	0/0/0/0
4	OCT	A	503	-	-	0/5/5/5	0/0/0/0
4	OCT	A	504	-	-	0/5/5/5	0/0/0/0
4	OCT	A	505	-	-	0/5/5/5	0/0/0/0
4	OCT	A	506	-	-	0/5/5/5	0/0/0/0

There are no bond length outliers.

There are no bond angle outliers.

There are no chirality outliers.

There are no torsion outliers.

There are no ring outliers.

1 monomer is involved in 2 short contacts:

Mol	Chain	Res	Type	Clashes	Symm-Clashes
3	A	502	1PE	2	0

5.7 Other polymers (i)

There are no such residues in this entry.

5.8 Polymer linkage issues (i)

There are no chain breaks in this entry.

CONFIDENTIAL VALIDATION REPORT

6 Fit of model and data (i)

6.1 Protein, DNA and RNA chains (i)

In the following table, the column labelled '#RSRZ> 2' contains the number (and percentage) of RSRZ outliers, followed by percent RSRZ outliers for the chain as percentile scores relative to all X-ray entries and entries of similar resolution. The OWAB column contains the minimum, median, 95th percentile and maximum values of the occupancy-weighted average B-factor per residue. The column labelled 'Q< 0.9' lists the number of (and percentage) of residues with an average occupancy less than 0.9.

Mol	Chain	Analysed	<RSRZ>	#RSRZ>2			OWAB(Å^2)	Q<0.9
1	A	403/408 (98%)	0.04	16 (3%)	42	51	26, 39, 67, 101	0

All (16) RSRZ outliers are listed below:

Mol	Chain	Res	Type	RSRZ
1	A	76	THR	5.6
1	A	225	THR	4.1
1	A	196	ALA	4.0
1	A	226	ILE	3.7
1	A	197	GLY	3.2
1	A	77	GLY	2.7
1	A	179	ASP	2.6
1	A	246	GLU	2.6
1	A	195	LEU	2.6
1	A	81	ALA	2.5
1	A	198	GLY	2.5
1	A	82	THR	2.4
1	A	230	ILE	2.4
1	A	229	GLY	2.2
1	A	249	ASP	2.2
1	A	329	TYR	2.1

6.2 Non-standard residues in protein, DNA, RNA chains (i)

There are no non-standard protein/DNA/RNA residues in this entry.

6.3 Carbohydrates (i)

There are no carbohydrates in this entry.

CONFIDENTIAL VALIDATION REPORT

6.4 Ligands (i)

In the following table, the Atoms column lists the number of modelled atoms in the group and the number defined in the chemical component dictionary. LLDF column lists the quality of electron density of the group with respect to its neighbouring residues in protein, DNA or RNA chains. The B-factors column lists the minimum, median, 95th percentile and maximum values of B factors of atoms in the group. The column labelled 'Q< 0.9' lists the number of atoms with occupancy less than 0.9.

Mol	Type	Chain	Res	Atoms	RSCC	RSR	LLDF	B-factors(Å^2)	Q<0.9
4	OCT	A	506	8/8	0.76	0.32	8.66	51,53,53,54	0
3	1PE	A	502	16/16	0.80	0.20	3.87	48,60,64,64	0
4	OCT	A	505	8/8	0.86	0.21	2.72	56,58,61,65	0
4	OCT	A	503	8/8	0.91	0.17	1.75	57,58,60,60	0
4	OCT	A	504	8/8	0.91	0.14	0.47	57,59,60,61	0
2	CA	A	501	1/1	0.99	0.07	-2.99	36,36,36,36	0

6.5 Other polymers (i)

There are no such residues in this entry.

CONFIDENTIAL VALIDATION REPORT
