# Supplementary material for: MOMP from Campylobacter jejuni Is a Trimer of 18-Stranded β-Barrel Monomers with a Ca2 + Ion Bound at the Constriction Zone
Source: J Mol Biol. 2016 Nov 6;428(22):4528–43. doi: 10.1016/j.jmb.2016.09.021 (PMC5090048; doi:10.1016/j.jmb.2016.09.021)
Supplement: Supplementary file 3 — Supplementary material 3. [file mmc3.pdf]

MOMP from *Campylobacter jejuni* is a trimer of 18 stranded β–barrel monomers with a metal ion bound at the constriction zone

Luana G M Ferrara[1], Gregor D Wallat[1], Lucile Moynié[1], Naresh N Dhanasekar[2], Soumeya Aliouane[3], Silvia Acosta-Gutiérrez[4], Jean-Marie Pagès[3], Jean-Michel Bolla[3], Mathias Winterhalter[2], Matteo Ceccarelli[4] & James H Naismith[1,5*]

[1]Biomedical Sciences Research Complex, University of St Andrews, St Andrews, UK

[2]Department of Life Sciences and Chemistry, Jacobs University Bremen, 28719 Bremen, Germany

[3]Aix Marseille Univ, IRBA, TMCD2, Marseille, France

[4]Department of Physics, University of Cagliari, Cittadella Universitaria Monserrato, S.P8-km 0.700,09042 Monserrato (CA), Italy

5 State Key Laboratory of Biotherapy, Sichuan University, Chengdu, China.

* To whom correspondence should be addressed naismith@st-andrews.ac.uk

SUPPORTING MATERIAL FOR PUBLICATION

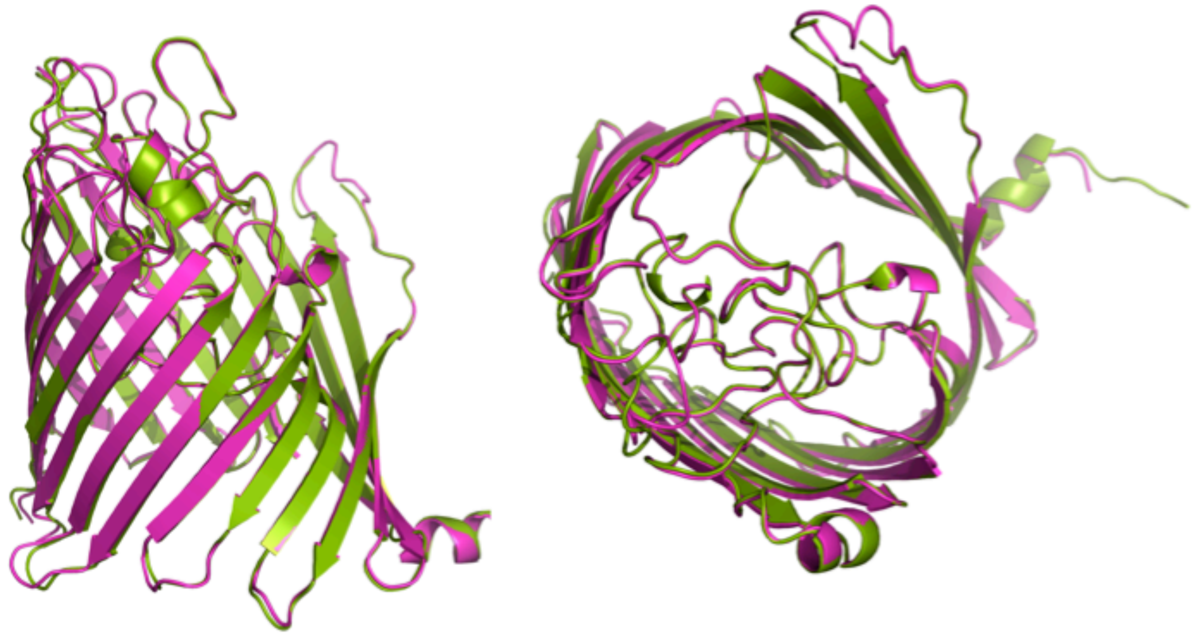

(a) (b)

Supplementary Figure 1. Superimposition of native MOMP (nMOMP shown in magenta) and the recombinant MOMP (rMOMP shown in green).

(a) View from the side.

(b) View from the outside the cell looking into the periplasm.

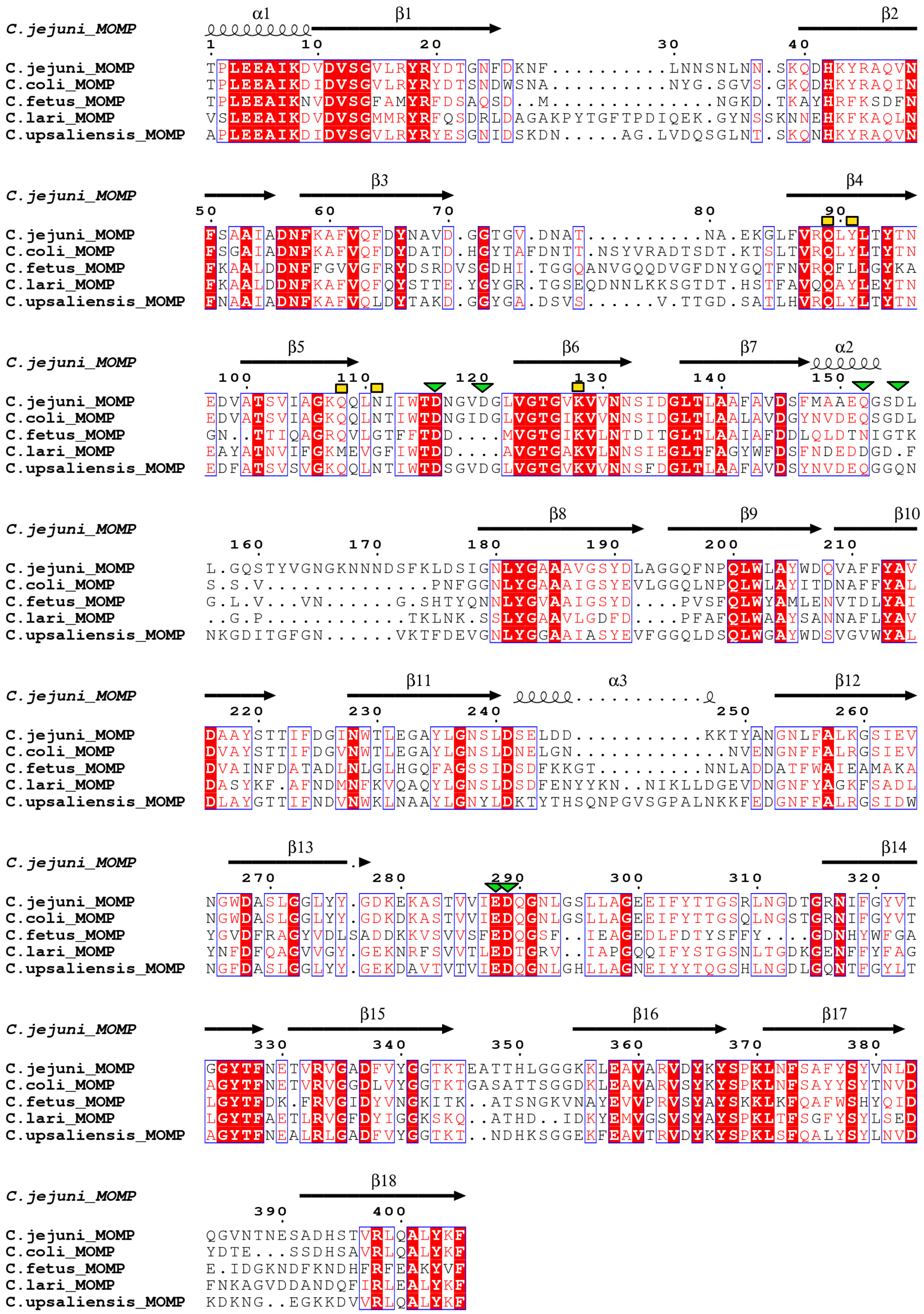

Supplementary Figure 2. Amino acid sequence alignment of MOMP from the five major campylobacter species. The green triangles highlight the conservation of the residues that are involved in metal binding. The yellow squares are residues in contact with PEG in the nMOMP structure.

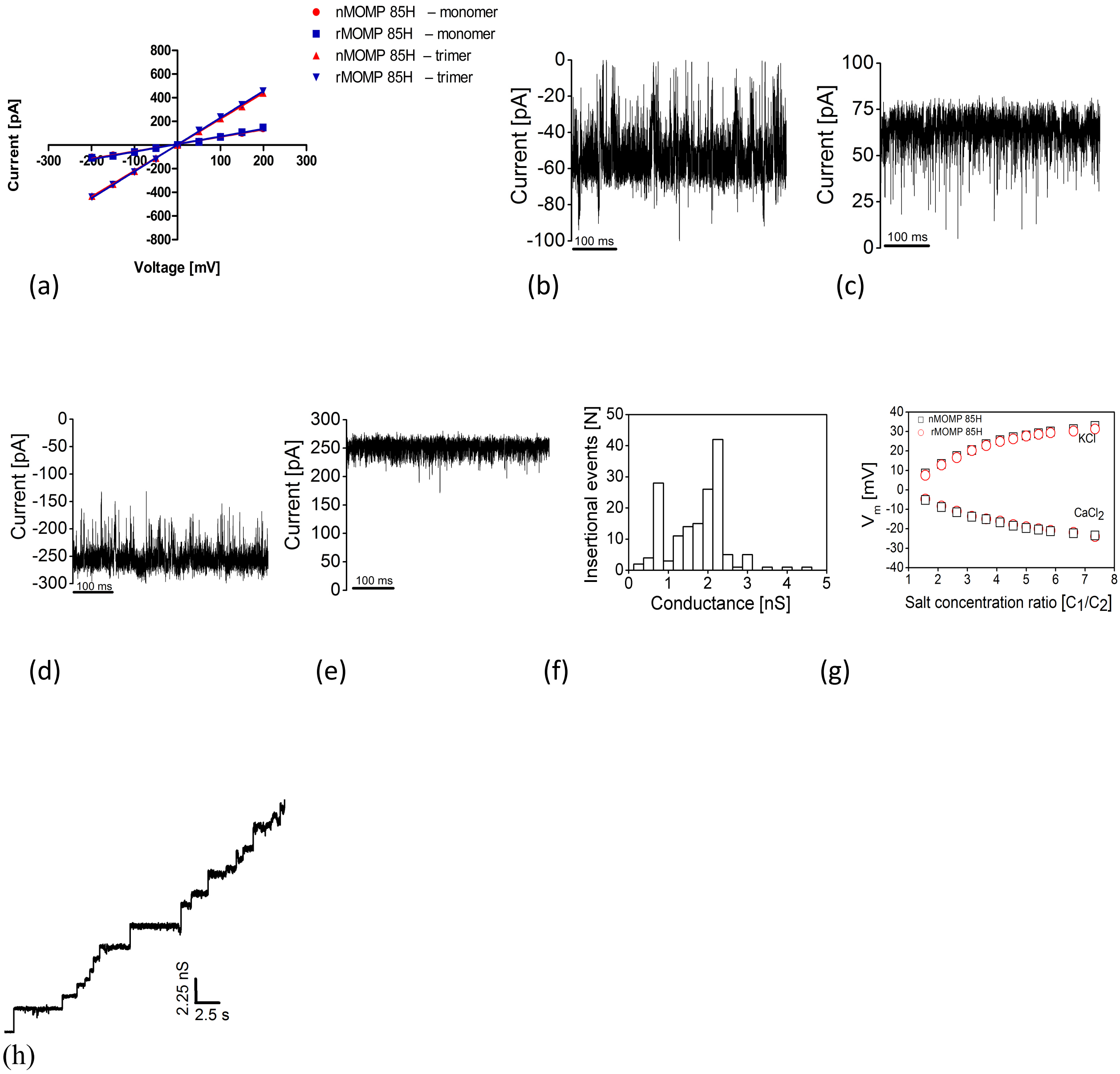

Supplementary Figure 3. Single channel behavior of MOMP

(a) Ion-current-voltage (I-V) relationship of the MOMP channel in 1M KCl 10 mM MES, pH 6.

(b) Electrical signature of a single nMOMP monomer in 1 M KCl, 10 mM MES, pH 6.0 at negative transmembrane potential of –100 mV.

(c) Electrical signature of a single nMOMP monomer in 1 M KCl, 10 mM MES, pH 6.0 at positive transmembrane potential of +100 mV.

(d) Ion-current trace of a single nMOMP trimer at negative transmembrane potential of –100 mV.

(e) Ion-current trace of a single nMOMP trimer at positive transmembrane potential of +100 mV.

(f) Histogram of the conductance steps observed after the addition of a single MOMP channel in 1 M KCl, 10mM MES at pH 6. The applied voltage was +20mV. 159 insertion events were recorded.

(g) Zero-current membrane potential (V_m) is plotted as a ratio of salt concentrations in both compartments for salt solutions ranging from 0.1 to 0.8 M KCl (for monovalent salt) and from 0.1 to 0.8 M $CaCl_2$ (for divalent salt).

(h) Multiple single channel insertions of the purified rMOMP in DPhPC/n-decane membranes bathed in 1M KCl, 10mM MES, pH 6.0. The applied voltage was +20 mV. The non uniform nature of multiple insertions of MOMP has been seen before [1].

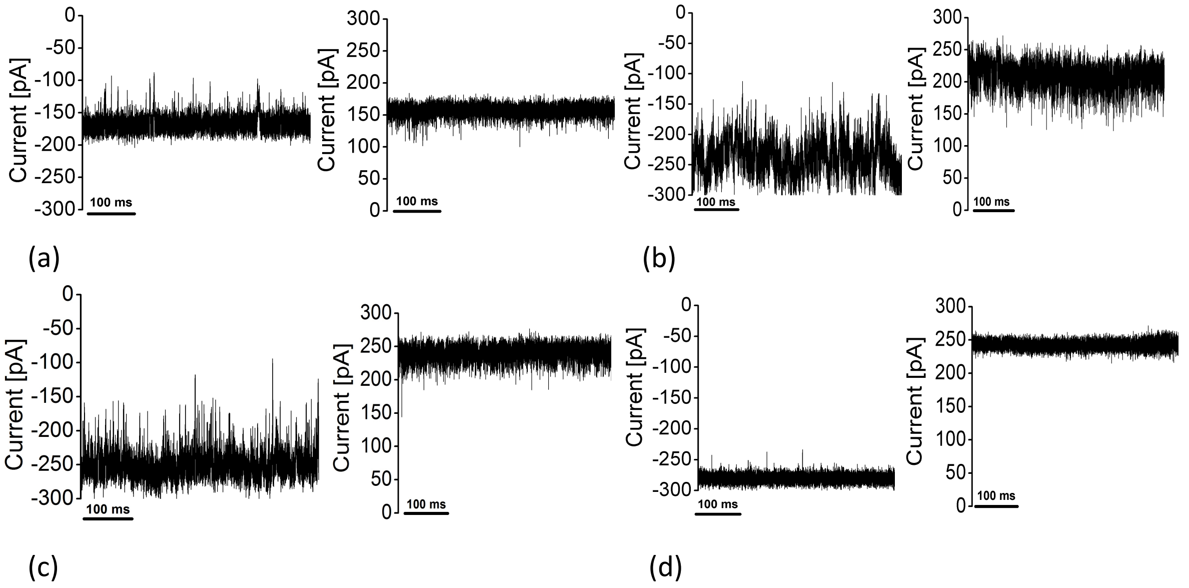

Supplementary Figure 4. Representative ion-current traces of rMOMP in 1M NaCl, 10 mM MES, pH 6.0.

(a) The ion-current corresponding to the trimer at negative (top panel, left) and positive (top panel, right) transmembrane potential of 100 mV.

(b) Single channel recordings of rMOMP in 1M KCl, 10 mM $ZnCl_2$, 10 mM MES, pH 6.0. The ion-current corresponding to the trimer at negative (top panel, left) and positive (top panel, right) transmembrane potential of 100 mV.

(c) Single channel recordings of rMOMP in 1M KCl, 10 mM MES, 10 mM EGTA at pH 6.0. The ion-current corresponding to the trimer at negative (bottom panel, left) and positive (bottom panel, right) transmembrane potential of 100 mV. The protein samples were incubated in 10 mM EGTA overnight prior carrying out single channel measurements.

(d) Single channel recordings of rMOMP after replacing the buffer used in (c) with1 M KCl, 10 mM MES, 10 mM $CaCl_2$ at pH 6.0. The ion-current corresponding to the trimer at negative (bottom panel, left) and positive (bottom panel, right) transmembrane potential of 100 mV.

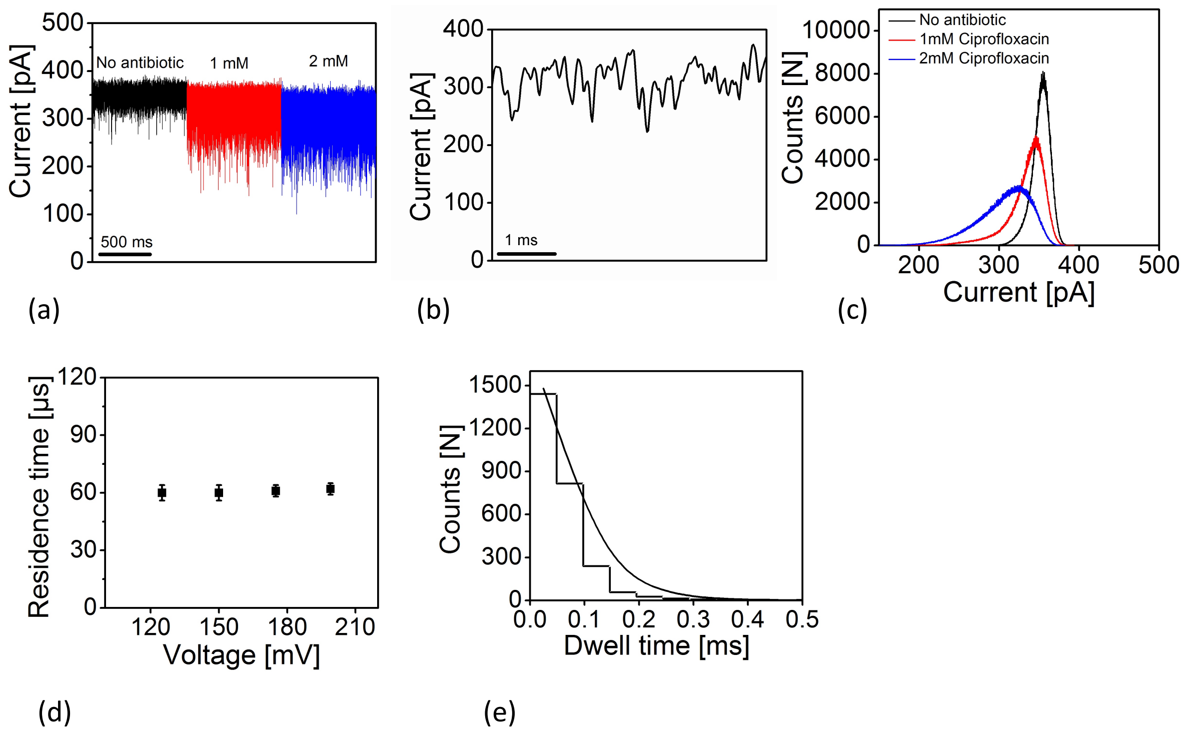

Supplementary Figure 5. Ciprofloxacin and MOMP in absence and presence of Ca^{2+}. Experimental conditions: 1 M KCl, 10 mM MES, pH 6.0 for (a–c); 1 M KCl, 10 mM MES, 10 mM $CaCl_2$, pH 6.0 for (d) and (e).

(a) Ion-current trace of a single rMOMP trimeric channel in the presence of ciprofloxacin, Ca^{2+} absent.

(b) The recordings with an expanded time scale of 2 mM ciprofloxacin with MOMP, Ca^{2+} absent.

(c) Corresponding current-amplitude histogram of the rMOMP channel with and without ciprofloxacin, Ca^{2+} absent.

(d) Residence time as a function of the applied voltage, Ca^{2+} present.

(e) Dwell-time histogram in the presence of 500 µM ciprofloxacin, Ca^{2+} present.

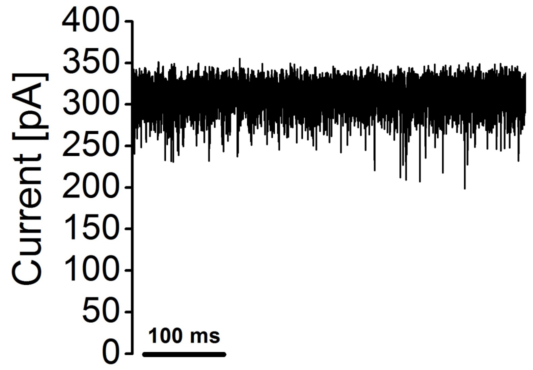

(a)

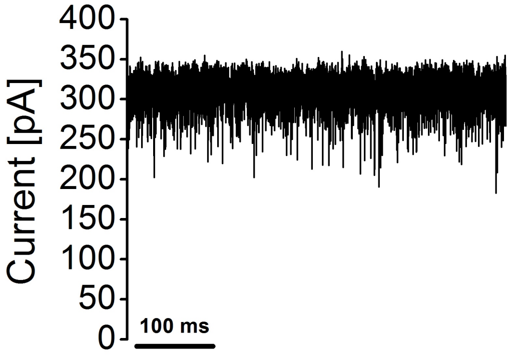

(b)

Supplementary Figure 6. The effect of Mg^{2+} on ciprofloxacin interaction with MOMP. Experimental conditions: 1 M KCl, 10 mM MES, 10 mM $MgCl_2$ pH 6.0. Voltage applied: + 150 mV
(a) Typical electrical signatures of the trimeric channel of rMOMP in the absence of ciprofloxacin
(b) Addition of 500 μM ciprofloxacin on the *trans* side.

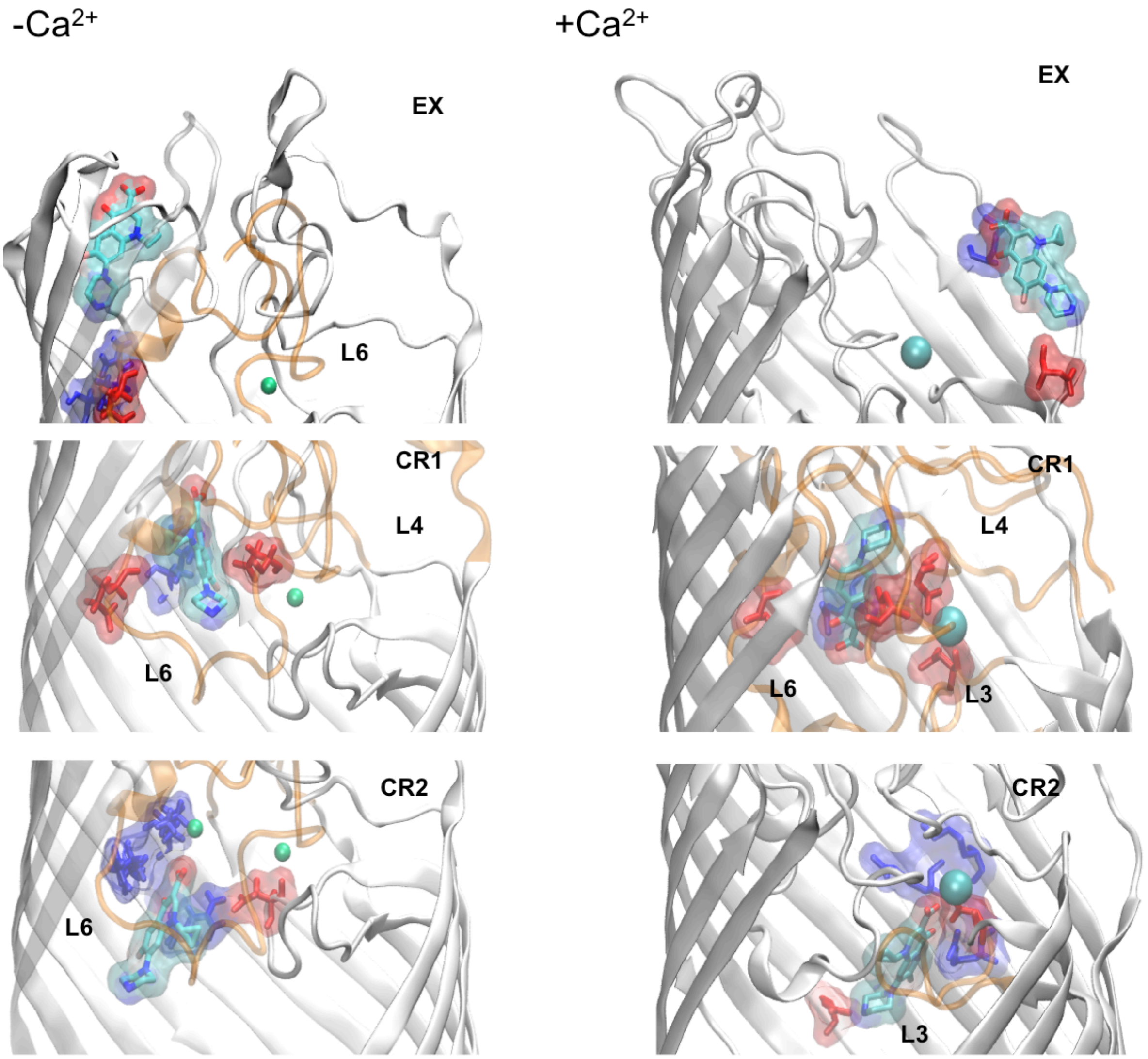

Supplementary Figure 7. Molecular dynamics of ciprofloxacin transport.
Most statistically relevant conformations for the chosen minima in Figure 5 are depicted EX, CR1 and CR2, for both scenarios. For clarity, β-strands from 9 to 14 has been removed from the cartoon representation of MOMP monomer. Ciprofloxacin is represented in licorice. Interacting loops are highlighted in orange and labeled. Specific charged residues interacting with ciprofloxacin are shown in licorice colored according to its charge.

Supplementary reference

[1] Page, W.J., Huyer, G., Huyer, M. & Worobec, E.A. (1989). Characterization of the porins of Campylobacter jejuni and Campylobacter coli and implications for antibiotic susceptibility. *Antimicrob Agents Chemother* **33**, 297–303.
(this is reference 35 in main text)
